# Global analysis of binding sites of U2AF1 and ZRSR2 reveals RNA elements required for mutually exclusive splicing by the U2- and U12-type spliceosome

**DOI:** 10.1093/nar/gkad1180

**Published:** 2023-12-13

**Authors:** Young-Soo Kwon, Sang Woo Jin, Hoseok Song

**Affiliations:** Department of Integrative Bioscience & Biotechnology, Sejong University, Seoul 05006, Korea; BK21 Graduate Program, Department of Biomedical Sciences, College of Medicine, Korea University Guro Hospital, Seoul 08308, Korea; BK21 Graduate Program, Department of Biomedical Sciences, College of Medicine, Korea University Guro Hospital, Seoul 08308, Korea

## Abstract

Recurring mutations in genes encoding 3′ splice-site recognition proteins, U2AF1 and ZRSR2 are associated with human cancers. Here, we determined binding sites of the proteins to reveal that U2-type and U12-type splice sites are recognized by U2AF1 and ZRSR2, respectively. However, some sites are spliced by both the U2-type and U12-type spliceosomes, indicating that well-conserved consensus motifs in some U12-type introns could be recognized by the U2-type spliceosome. Nucleotides flanking splice sites of U12-type introns are different from those flanking U2-type introns. Remarkably, the AG dinucleotide at the positions −1 and −2 of 5′ splice sites of U12-type introns with GT-AG termini is not present. AG next to 5′ splice site introduced by a single nucleotide substitution at the −2 position could convert a U12-type splice site to a U2-type site. The class switch of introns by a single mutation and the bias against G at the −1 position of U12-type 5′ splice site support the notion that the identities of nucleotides in exonic regions adjacent to splice sites are fine-tuned to avoid recognition by the U2-type spliceosome. These findings may shed light on the mechanism of selectivity in U12-type intron splicing and the mutations that affect splicing.

## Graphical Abstract

**Graphical Abstract:**
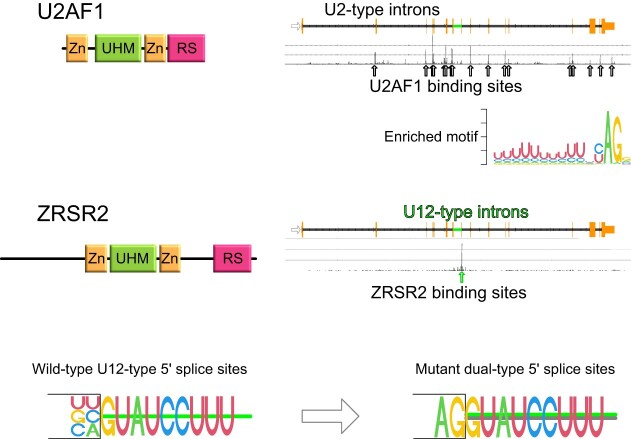

## Introduction

Most human pre-mRNAs are interrupted by U2-type introns that predominantly begin with the GU dinucleotide and end with the AG dinucleotide as parts of quite degenerate longer consensus sequences covering exon-intron boundary regions. U2-type introns are recognized and excised by a large and dynamic protein-RNA complex, the spliceosome, which contains U1, U2, U4, U6 and U5 small nuclear RNAs (snRNA). Base pairing of U1 snRNA with the 5′ splice site and base pairing of U2 snRNA with the branch point sequence (BPS) are required for the splicing of U2-type introns (1). An adenosine residue in the BPS forms a 2′ to 5′ phosphodiester bond with the 5′ end of the intron at the first catalytic step of the splicing reaction. The 3′ AG dinucleotide is involved in marking the boundaries between introns and 3′ exons and in exon ligation at the second catalytic step of the splicing reaction. Base pairing of U2 snRNA with the degenerate BPS is facilitated by the highly conserved U2AF heterodimer composed of the large subunit of the U2 auxiliary factor (U2AF2/U2AF65) and the small subunit (U2AF1/U2AF35). U2AF2 binds the polypyrimidine tract (PPT) located between the BPS and the 3′ splice site. U2AF1 contacts both the 3′ terminal AG dinucleotide and the immediately downstream exonic region, which may play an essential role in defining the 3′ splice site containing a weak PPT at an early step of spliceosome assembly before the first catalytic step (2–4). Because of recurring somatic mutations in U2AF1 in hematological malignancies, there are increasing interests in the functions of the splicing factor in both physiological and pathological settings (5). Intriguingly, the human genome encodes U2AF1-related splicing factors including U2AF26, ZRSR1 (U2AF1-RS1) and ZRSR2 (U2AF1-RS2/Urp) that bear sequence similarities to U2AF1 (6–8). Biochemical studies suggested that U2AF1 and ZRSR2 have distinct functions in splicing U2-type introns since U2AF1 could not restore U2-type intron splicing in ZRSR2-depleted extracts (8).

In contrast to the extremely prevalent U2-type introns, rare U12-type introns are present in a small number of genes (9,10). U12-type introns have well-conserved consensus sequences at the 5′ splice site (RUAUCCUU) and the BPS (UCCUURAY) (9), which have been used for the computational identification of U12-type introns (11). In addition, a multitude of U12-type introns lack the PPT and the termini of U12-type introns are often occupied by the AU-AC dinucleotide pair. For the excision of U12-type introns from pre-mRNAs, a specialized U12-type spliceosome comprising U11, U12, U4atac, U6atac and U5 snRNPs is required. The functions of U11, U12, U4atac and U6atac snRNPs correspond to those of U1, U2, U4 and U6 snRNPs in the U2-type spliceosome, respectively. Although U12-type introns have tightly constrained consensus sequences, the extent to which deviation from consensus sequences is tolerated is not firmly established. Besides, the degeneracy of the U2-type consensus sequences makes it difficult to distinguish U12-type introns from U2-type introns showing sequence properties of U12-type introns. Moreover, it is not established whether introns can be excised by both U2-type and U12-type spliceosomes in the cell. Considering the high fidelity in selecting the correct splice site pair, a fundamental question is how the U2- and U12-type spliceosomes have such exceptional capacity to select correct splice sites among numerous pseudo and cryptic sites.

For all rarity of U12-type introns in the genome and the minor spliceosome, genetic or somatic mutations in genes encoding components specific to the U12-type spliceosome have been associated with multiple diseases, including developmental disorders, neurodegeneration and cancer, signifying the importance of U12-type intron splicing in human physiology and pathology (12–14). Given the high information content of longer constrained U12-type consensus sequences, the absence of the PPT and the frequent presence of nucleotides other than G at the 3′ terminal position, the U2AF heterodimer may be dispensable for splicing of U12-type introns. Consistent with this possibility, U2AF-independent splicing of U12-type introns was demonstrated in biochemical studies (15). In contrast, ZRSR2 has been shown to be indispensable for splicing of U12-type introns (14,16). Nonetheless, the exact role of ZRSR2 in RNA splicing of U2-type and U12-type introns remains largely unclear.

Since somatic mutations have been frequently found in *ZRSR2* gene in hematological malignancies (5), there is growing interest in better understanding of the functions of U2AF1-relaed factors in splicing of U2-type and U12-type introns. U2AF1 and U2AF26 appear to have identical *in vitro* activity in U2-type intron splicing, as U2AF26 can functionally substitute for U2AF1 in splicing (7). In contrast, the paralogous splicing factors ZRSR1 and ZRSR2 that are 94% identical to each other have been regarded to play essential roles in splicing of both U2-type and U12-type introns (16,17). ZRSR1 and ZRSR2 genes are required for viability of human cells, yet mouse orthologous genes are functionally redundant to each other (16,18), which impose difficulties on functional studies of the ZRSR1/2 factors in the cell. Because biochemical and genetic approaches have long been used to study functions of U2AF1 and U2AF1-related proteins, remaining questions might be difficult to be addressed by using similar approaches. We reasoned that defining the RNA interactomes might help address important questions on the functions of these RNA-binding proteins and provide insights into the molecular mechanisms underlying their functions.

Here, we determined the *in vivo* binding profiles of U2AF1 and ZRSR2 in human embryonic stem cells (ESC) by crosslinking-immunoprecipitation coupled with next-generation sequencing (CLIP-seq). Since UV induces crosslinking exclusively between protein and RNA in proximity, CLIP is particularly useful to investigate *in vivo* binding sites of RNA binding proteins. Unexpectedly, the binding sites of U2AF1 and ZRSR2 show that the previously defined U12-type consensus sequence motifs are necessary but not sufficient for RNA splicing mediated by the U12-type spliceosome, and that a small number of naturally occurring 3′ slice sites containing the U12-type motifs are spliced by both the U2-type and U12-type spliceosomes. Since most introns containing U12-type consensus sequence motifs are not excised by both the U2-type and U12-type spliceosome, there should be additional elements that further specify which spliceosome removes the intron. We conclude that 5′ exonic nucleotides immediately upstream of an intron play an important role in selection of the appropriate spliceosome class for the intron.

## Materials and methods

### Insertion of a C-terminal HA-tag at the ZRSR2 genomic locus

H1 hESCs were maintained on feeder plates as previously described (19). Knock-in of a tag comprising three copies of the HA-tag was carried out via CRISPR/Cas9-induced cleavage and homology-dependent DNA repair. sgRNA was designed to a target downstream region of the ZRSR2 stop codon. To generate the targeting construct, a genomic region encompassing the last codon of ZRSR2 was PCR amplified and inserted into pBluescript II SK2(+). The target sequence of the sgRNA was removed, and the stop codon was replaced with the HA-IRES-puro cassette (Supplementary Figure S1A).

The plasmid/lipid solution was prepared with 1 μg of pRGEN-CMV-Cas9 (ToolGen), 0.5 μg of a guide plasmid and 0.5 μg of the targeting construct using Lipofectamine 3000 as a transfection reagent. Human ESCs (500 000) were dissociated into single cells by trypsinization, washed once with OPTI-MEM (Thermo) and resuspended in 100 μl of the plasmid/lipid solution. The cell suspension was incubated for 15 min at room temperature before plating on feeder plates with growth medium supplemented with 10 μM of the ROCK inhibitor (Y-27632). After 48 hrs, the transfected cells were maintained with growth medium containing 0.5 μg/ml puromycin. The gene targeting in the puromycin-resistant clones was verified by western blot using anti-HA antibodies and sequencing cDNA. All tested puromycin-resistant clones expressed the HA-tagged ZRSR2 (Supplementary Figure S1B).

### In vivo analysis of splicing using minigene constructs

Genomic loci flanking the introns of interest in *RBFOX2*, *CNBP*, *EXOSC1*, *RABGGTA* and *SBNO1* were PCR amplified with phosphorylated primers using Phusion DNA polymerase (NEB) and cloned into the HindIII/XbaI sites of pcDNA3.1. The restriction enzyme sites were treated with calf intestinal phosphatase and blunt-ended by T4 DNA polymerase. Schematics of the minigene constructs and primer sequences used for cloning and for introduction of mutations are in the Supplementary materials (Supplementary Figure S2 and Supplementary Table S1).

For *in vivo* analysis of splicing, HeLa cells were transfected with the minigene constructs using Lipofectamine 3000 and harvested after 24 hrs. cDNA produced from 2.5 ng of total RNA was PCR amplified using the T7 and BGH reverse primers for 25 cycles at 95°C for 15 s, 55°C for 15 s and 72°C for 30 s. The PCR products were separated according to size using the Qsep100 DNA analyzer (BiOptic).

### CLIP experiments

CLIP-seq was performed based on a previously described protocol with some modifications (20,21). H1 human ESCs were maintained and passaged twice on Matrigel-coated plates with MEF-conditioned media supplemented with 50 ng/ml bFGF before irradiation with 300 mJ/cm^2^ of UVC light (254 nm) using the CL-1000 ultraviolet crosslinker (UVP). Two million cells were resuspended in 250 μl lysis buffer (50 mM Tris–HCl pH 7.4, 100 mM NaCl, 1 mM MgCl_2_, 0.1 mM CaCl_2_, 1% NP-40, 0.5% sodium deoxycholate, 0.1% sodium dodecyl sulphate) containing ethylenediaminetetraacetic acid (EDTA)-free protease inhibitor cocktail (Roche) and RNase inhibitor (Ambion) and briefly sonicated using the Bioruptor. The lysates were treated with 5 μl of Turbo DNase (Ambion) and 10 μl of 10 U/μl or 1 U/μl RNase I (Ambion) at 37°C for 3 min. Cellular debris was removed by centrifugation at 13000 rpm for 15 min. Protein–RNA complexes were immunopurified with a mixture of anti-U2AF1 antibodies (Bethyl Laboratories, A302-079, A302-080) or an anti-HA antibody bound to Protein A/G Dynabeads (Supplementary Figure S3A and B). Beads were washed twice with high-salt wash buffer (50 mM Tris–HCl pH 7.4, 1 M NaCl, 1 mM EDTA, 1% NP-40, 0.5% sodium deoxycholate and 0.1% SDS) followed by two washes with PNK buffer (20 mM Tris–HCl pH 7.4, 10 mM MgCl_2_ and 0.2% Tween-20). For dephosphorylation of 3′ ends, 1 U of FastAP alkaline phosphatase (Fermentas) was added to the beads and the beads were incubated at 37°C for 20 min. After washing the beads twice with PNK buffer, protein-bound RNAs were ligated to a pre-adenylated 3′ adapter overnight at 16°C in RNA ligase buffer containing 25% PEG6000 by using 12.5 U of T4 RNA ligase 1 (NEB) and 25 U of T4 RNA ligase 2, truncated K267Q (NEB). Beads were washed twice with PNK buffer. The protein-bound RNAs were separated using a 4–12% NuPAGE Bis-Tris gel (Invitrogen), transferred to a nitrocellulose membrane and excised from the membrane. The protein was degraded by proteinase K treatment, and RNA was recovered by phenol/chloroform/isoamyl alcohol extraction and ethanol precipitation.

### Sequencing library construction

Sequencing library preparation was performed according to a previously described protocol (20). Protein-associated RNAs purified by CLIP were ligated to a pre-adenylated 3′ adaptor that contains a 2-nt adapter code for de-multiplexing and a 3-nt random sequence to minimize the loss of information after collapsing identical sequences. The RNA-adapter hybrids were converted into cDNAs with reverse transcriptase (RT) primer and SuperScript III RT (Invitrogen). The cDNAs were size fractionated by electrophoresis on a 12% denaturing polyacrylamide gel, eluted from the gel and circularized by single-stranded DNA ligase (Epicentre). Circularized cDNAs were amplified with PCR primers containing a 6-nt primer code for de-multiplexing. PCR products were purified by agarose gel elution and subjected to next-generation sequencing by using the Illumina NEXTSeq 500 platform. Primer and adaptor sequences are detailed in Supplementary Table S1.

### Read processing and mapping

Multiplexed raw sequence reads were de-multiplexed using an adapter and primer codes, and adapter-derived sequences were trimmed before further analysis of sequences using functions, such as trimLRPatterns, in a Bioconductor package, ShortRead (22). Because the reads are complementary sequences of fragmented RNAs, the reads were converted into complementary sequences by using the reverseComplement function and collapsed before mapping to database sequences using the occurrenceFilter function in the ShortRead package. The processed reads mapped to abundant noncoding RNA sequences using the Bowtie short read aligner (version 1.0.1) with parameters -q -m 50 -n 2 -l 70 -5 1 -3 5 -a –best –strata -p 11 –sam –norc. For mapping to rRNA regions, the sense strand of U13369 rDNA sequence was used. Sense-strand sequences of 5S rRNA, snRNA, snoRNA, tRNA and miscellaneous non-coding RNAs were retrieved from the human hg18 reference genome by using coordinates obtained from the UCSC genome browser by using table browser. Reads mapped to pre-miRNA using sense-strand sequences obtained from the miRBase database (release v20). For peak calling, reads were uniquely mapped to the human reference genome (GRCh37/hg19) without allowing any mismatch were used for peak calling to minimize alignment artefacts. Categorization and quantification of reads uniquely mapping to the human genome were conducted by using functions in the GenomicFeatures package and the TxDb.Hsapiens.UCSC.hg19.knownGene annotation package for human transcripts. Resizing and shifting of coordinates of aligned reads and defined peaks were conducted by using functions in a Bioconductor package, GenomicRanges. Data visualization was performed by using functions in R/Bioconductor packages, ggplot2, ggbio (23) and ChIPseeker (24). MEME motif enrichment analysis (http://meme-suite.org/meme/tools/meme) was conducted to find enriched motifs (6–15 motif width and the zoops options). A Bioconductor package, seqPattern (25), was used to visualize the positions of nucleotides.

## Results

### U2AF1 mainly binds non-functional sites in introns and functional 3′ splice sites

To map U2AF1 binding sites in human ESCs, we used a CLIP-seq approach with a mixture of two anti-U2AF1 antibodies that do not work individually. For pinpointing binding sites of RNA binding proteins, short uniquely mappable sequencing read covering a complete RNA fragment is more useful in determining protein binding sites than a read covering part of a long RNA fragment. We used two different RNase treatment conditions with two different biological replicates to generate four independent libraries, in which average insert sizes were shorter than 20 nt. In addition, we generated a negative control library by CLIP procedure with a non-specific IgG antibody. After adapter trimming and collapsing of identical sequencing reads, we combined 65953372 unique reads containing an insert longer than 18 nt from four highly reproducible libraries (Supplementary Figure S4).

**Figure 1.**
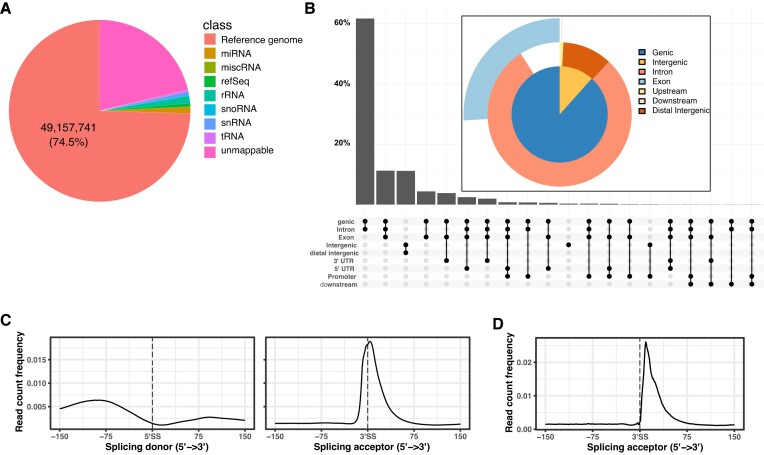
U2AF1 binds 3′ splice sites and non-functional sites in pre-mRNA. (**A**) Distribution of U2AF1 CLIP reads on the genome. The pie chart shows the percentages of collapsed reads mapping to non-coding RNAs and other regions in the UCSC human reference genome (GRCh37/hg19). The vast majority of mappable reads are located in genomic regions other than abundant non-coding RNAs. See also Supplementary Figure S4 for detailed information about the numbers of reads from experimental replicates and reads mapping to different classes of sequences. (**B**) UpSet plot and pie chart showing U2AF1 binding to introns and exon-intron junctions. The UpSet plot and the pie chart show the distribution of reads uniquely mapping to the human reference genome in the intergenic, intronic, coding regions and 5′ and 3′ UTRs. (**C**) Coverage profile of U2AF1 CLIP reads around splice sites. The CLIP read coverage profile relative to 3′ splice sites shows that U2AF1 directly contacts narrow regions peaking at the 3′ splice sites. (**D**) Distribution of 3′ ends of CLIP reads. The absence of 3′ ends of CLIP reads in the intronic region shows that U2AF1 does not contact regions upstream of 3′ splice sites.

For the identification of classes of interacting RNAs, we aligned CLIP reads to seven classes of sequences (rRNAs, snRNAs, snoRNAs, tRNAs, miscellaneous RNAs, microRNAs and the UCSC hg19 reference human genome). U2AF1 CLIP-seq reads mostly aligned with regions of the reference genome other than non-coding RNA genes, while abundantly expressed functional noncoding RNAs such as rRNAs, tRNAs and snRNAs contributed only 4.6% of the CLIP reads, signifying specificity of U2AF1 CLIP-seq (Figure 1A). The vast majority (87%) of reads uniquely mapping in the UCSC hg19 reference genome were in annotated known genic regions (Figure 1B).

We detected the majority of contacts between U2AF1 and pre-mRNA in intronic regions away from the annotated 3′ splice sites, which is consistent with previous genome-wide analyses of U2AF2 binding showing numerous binding events beyond genuine 3′ splice sites such as antisense L1 repeats (26,27). While CLIP reads overlapping 25 bp exonic regions beginning from 3′ splice sites of annotated UCSC hg19 known genes, which constitute 0.4% of annotated genic regions, accounted for 18.3% of reads mapping in genic regions, reads overlapping 25 bp exonic regions ending at 5′ splice sites contributed just 2.5% of reads mapping in the genic regions, signifying that CLIP for U2AF1 specifically enriched 3′ splice sites in known genic regions.

The CLIP read coverage profile relative to 3′ splice sites shows that U2AF1 directly contacts narrow regions peaking at the 3′ splice sites (Figure 1C). Notably, the vast majority of 3′ ends of reads were located in downstream regions of 3′ splice sites and few 3′ ends of reads were found in introns next to 3′ splice sites (Figure 1D). The positions of 3′ ends of reads relative to 3′ splice sites indicate that U2AF1 should mainly contact nucleotides at 3′ splice sites and immediately downstream regions of pre-mRNA. In contrast to mapping to 3′ splice sites, reads mapping across introns were extremely rare, indicating that the majority of U2AF1 should leave pre-mRNA before completion of the second step of splicing reaction. These results are in line with biochemical studies demonstrating that U2AF1 contacts the AG dinucleotide at the 3′ splice site and the immediate downstream region of an adenovirus major late pre-mRNA derivative in an AG-dependent manner (2) and U2AF1 leaves the spliceosome before the second catalytic step of splicing (16).

### U2AF1 mainly binds the consensus sequence of the U2-type 3′ splice site

The requirement of AG for *in vitro* crosslinking of U2AF1 to 3′ splice sites (2) raised the question of whether AG is essential for the *in vivo* interactions of U2AF1 with 3′ splice sites of naturally occurring introns and U2AF1 binding sites in deep intronic regions. To investigate nucleotides involved in U2AF1 binding *per se*, we divided U2AF1 binding sites based on their overlaps with annotated 3′ splice sites and analyzed nucleotide occurrences separately. To define robust binding sites, we selected the genomic regions wider than 9 nucleotides uniquely covered at least 20 times by U2AF1 CLIP reads. U2AF1 binding peaked at 4 nucleotides downstream of 3′ splice sites (Figure 1C) and maximally covered positions were not exactly located at the centers of defined U2F1 binding sites. We set 50-nt-long windows around the maximally covered positions, assuming that the peak positions are crosslinked sites. The 50-nt-long windows instead of regions above the threshold were used to categorize corresponding U2AF1 binding sites into 53929 sites overlapping annotated 3′ splice sites and 108739 binding sites that are away from annotated 3′ splice sites.

**Figure 2.**
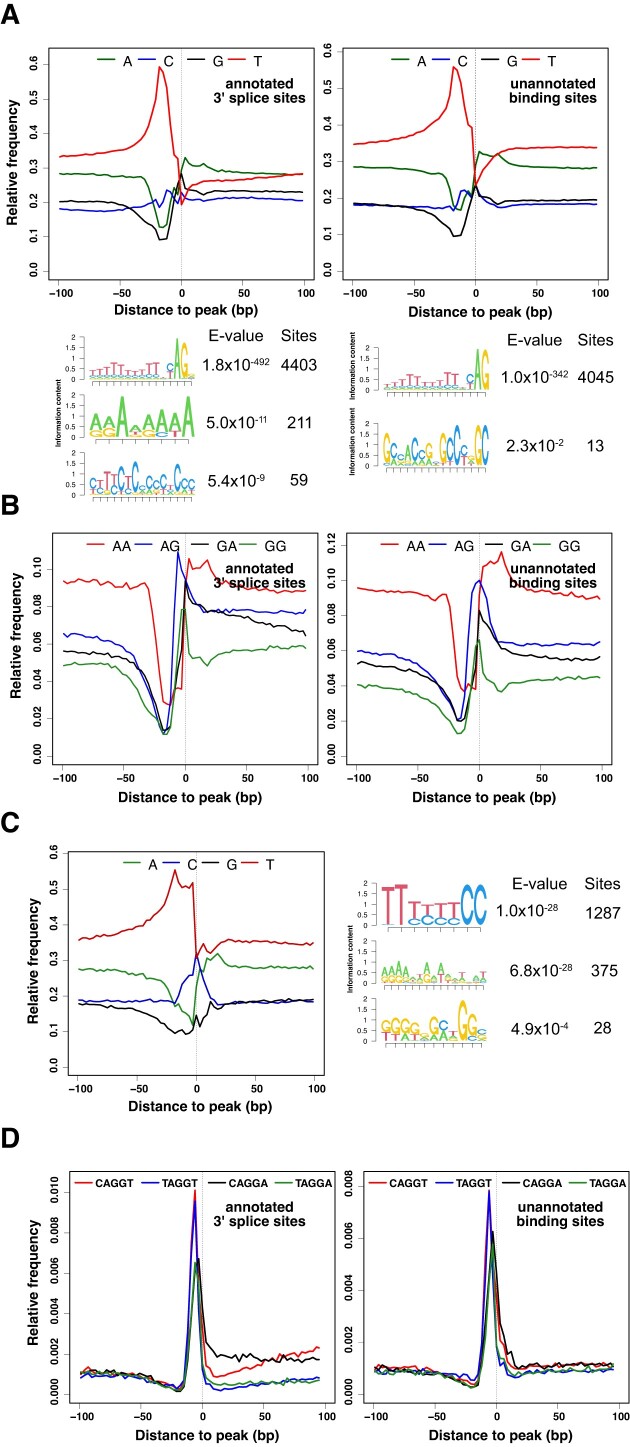
U2AF1 binds the 3′ splice site consensus. (**A**) Frequency of single nucleotides relative to U2AF1 binding peaks. For motif enrichment analysis, 5000 strongest peaks were chosen from the repeat masked human genome sequence and 50-nt sequences upstream of peaks were used for motif enrichment analysis. (**B**) Frequency of dinucleotides relative to U2AF1 binding peaks. The AG dinucleotide frequency is highest at peaks of U2AF1 binding. (**C**) Frequency of single nucleotides relative to U2AF1 binding peaks in AG-less sites. Pyrimidine-rich regions of AG-less sites are wider than those of U2AF1 binding sites having the AG dinucleotide. For motif enrichment analysis, 5000 50-nt sequences chosen from the strongest peaks aligned to the masked human genome were analyzed. (**D**) Frequency of pentamer motifs relative to U2AF1 binding peaks. The most frequent motifs found at 3′ splice sites are concentrated not only at the peaks overlapping 3′ splice sites but also at the peaks located in unannotated intronic regions.

The analysis of single nucleotide occurrences showed that U2AF1 binding sites not only at annotated 3′ splice sites but also in deep intronic regions had U-rich/purine-depleted tracts located immediately upstream of peaks of U2AF1 CLIP read coverage (Figure 2A). U-content was lowest at the peak positions and lower in downstream regions of U-rich tracts than in upstream regions, which is also found at annotated splice sites (Supplementary Figure S5). In contrast, A- or G-content was highest at around peaks.

We surveyed over-represented motifs upstream of peaks overlapping 3′ splice sites and located in introns, respectively. The most significantly enriched motifs were surprisingly identical, indicating that a lot of pseudo splice sites are recognized by U2AF1. The fractions of U2AF1 binding sites bearing an AG dinucleotide within the 20 nt of peaks were significantly high (97.0% and 84.1% for binding sites overlapping annotated 3′ splice sites and intron regions only, respectively), considering that just 61.2% and 58.1% of shuffled sequences were expected to bear the AG dinucleotide in the same regions (Figure 2B). Although the high fraction of sequences containing the AG dinucleotide is consistent with *in vitro* crosslink studies that demonstrated specific contacts between U2AF1 and the AG dinucleotide (2,3), a non-negligible fraction (15.9%) of U2AF1 binding sites in intronic regions did not have the AG dinucleotide, indicating that U2AF1 could bind regions lacking the AG dinucleotide in an unproductive manner. To investigate the characteristic RNA elements located in AG-less U2AF1 binding sites, we analyzed nucleotide occurrences in AG-less sites. The analysis of single nucleotide occurrences showed that long pyrimidine-rich tracts, each of which consisted of a U-rich region followed by a short C-rich region, extended up to peaks of U2AF1 binding (Figure 2C). The enrichment of pyrimidine-rich regions at AG-less regions by U2AF1 CLIP is consistent with previous observations that the early steps of *in vitro* spliceosome assembly prior to catalysis at splice sites with long pyrimidine tracts are independent of the AG dinucleotide and U2AF1 (2,28).

In addition to AG at the 3′ end of the intron, the identity of the first nucleotide of the 3′ exon was demonstrated to be an important determinant of U2AF1-mediated activation of *in vitro* splicing of AG-dependent introns (29). The AG dinucleotide of the AG-dependent 3′ splice site is recognized both at the early spliceosome assembly step by U2AF1 and at the second catalytic step of splicing. Therefore, the bias for certain sequence motifs encompassing AG at the 3′ splice site may be attributed either to preferential binding of U2AF1 to the motifs at the early stage of splicing or to higher catalytic activity of the spliceosome toward 3′ sites containing the motifs at the second catalytic step. To test whether nucleotides flanking AG influence U2AF1 binding *per se*, we analyzed motif occurrences in binding sites at annotated 3′ splice sites and in intronic regions.

The analyses of trinucleotide occurrences revealed that CAG, AGG and UAG were the most to the third most frequently occurring motifs at peaks of U2AF1 binding in both annotated 3′ splice sites and in intronic regions (Supplementary Figure S6). The analysis of tetranucleotide occurrences showed that CAGG, UAGG and AGGU were the most significantly enriched motifs from U2AF1 binding sites and AGGA was also a significantly enriched motif (Supplementary Figure S7). The analysis of pentanucleotide occurrence showed that CAGGU and UAGGU were the most enriched motifs from U2AF1 binding sites overlapping annotated splice sites or unannotated intronic sites and CAGGA and UAGGA were the next most enriched motifs (Figure 2D). The most frequently found motifs from U2AF1 binding sites indicate that the bias for G at the + 1 position is attributed to preferential binding of U2AF1.

In contrast to motifs most frequently found in U2AF1 binding sites, CAGGU and CAGGA are the most and second most common motifs at the annotated 3′ splice sites retrieved from the reference human genome (Supplementary Figure S8), indicating that U preceding AG is less preferred in naturally occurring splice sites. Interestingly, CAGGG and CAGGC were more frequently found motifs in both U2AF1 binding sites located at annotated 3′ splice sites and unannotated intronic regions than UAGGG and UAGGC motifs, (Supplementary Figure S9), suggesting that the C nucleotide at the -3 position of the 3′ splice site may help U2AF1 bind to suboptimal sequences. Taken together, these data show that U2AF1 preferentially binds sequences similar to the consensus sequence motif of annotated 3′ splice sites.

### U2-type AG-less splice sites are independent of downstream AG

U2AF1 binding to pyrimidine-rich AG-less sites in the cell raised the possibility that the strict requirement of AG for the second catalytic step of *in vivo* pre-mRNA splicing might mainly impose the tight conservation of the AG dinucleotide at the 3′ splice site. However, the AC and UG dinucleotides are located at 3′ ends of a small number of U2-type introns (30,31), demonstrating that AG is not always required for U2-type intron splicing. To better understand the requirement for the 3′ AG dinucleotide at different steps in splicing, we investigated U2AF1 binding to AG-less splice sites that constitute 0.2% of annotated U2-type introns. U2AF1 CLIP reads overlapping AG-less splice sites accounted for 0.06% of reads overlapping the annotated 3′ splice sites. Remarkably, 90% of reads overlapping an AG-less splice site also overlapped an immediately downstream in-frame alternative splice site containing the AG dinucleotide (Supplementary Table S2). Since splicing patterns and sequences flanking these deviant 3′ splice sites are conserved across orthologous mammalian genes (30), these in-frame alternative splice sites point to biological relevance rather than spurious splicing. In addition to these alternative splice sites, constitutive 3′ splice sites of U2-type introns ending with extremely rare AU-AC termini are followed by evolutionarily conserved out-of-frame 3′ AG dinucleotides. These conserved unusual splice site structures prompted us to speculate that the downstream AG dinucleotide might be required for splicing to the upstream non-canonical splice sites.

**Figure 3.**
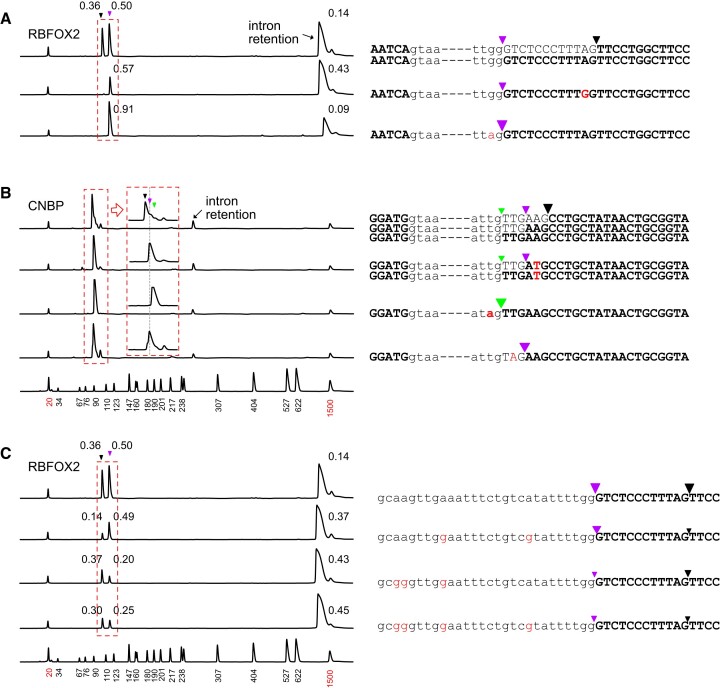
AG-independent splicing at AG-less splice sites. Effects of mutations at 3′ splice sites of (**A**) *RBFOX2* and (**B**) *CNBP* mini-gene constructs on alternative splicing. Transcripts from mini-gene constructs were amplified by RT-PCR and separated by a capillary fragment analyzer. Splice sites are presented on the left of graphs of DNA fragment distribution. The splice sites determined by DNA sequencing are marked by inverted triangles and spliced RNAs in the red dotted rectangles are represented by bold letters. Annotated intron and exon sequences are represented in lower-case and upper-case letters, respectively. The red letters denote the nucleotide substitutions. The numbers above peaks show relative intensities normalized by the lengths of peaks. The structures of mini-genes are shown in Supplementary Figure S2. (**C**) Effect of BPS mutations on splicing patterns at the alternative sites of *RBFOX2*. Changes in alternative site selection were observed when at least two predicted BPSs were mutated simultaneously. The lowest graphs in (B) and (C) show the positions of size markers.

To investigate the role of dinucleotides at these unusual alternative splice sites, single nucleotide substitutions were introduced into mini-gene constructs derived from the *RBFOX2* and *CNBP* genes to convert canonical splice sites to non-canonical sites and vice versa (Supplementary Figure S2). Mini-gene constructs containing the individual mutations were transfected into Hela cells and the patterns of spliced RNA products from the transfected expression constructs were determined using reverse transcription followed by PCR amplification. The splice sites of RNAs transcribed from the wild-type and mutated constructs are shown in Figure 3A. Consistent with previous observations, all mutations changing downstream 3′ AG dinucleotides to GG and UG dinucleotides completely blocked splicing to the mutated 3′ sites, resulting in increased retention of the intron in *RBFOX2*. The inactivating mutations, however, did not inhibit or enhance splicing to upstream AG-less 3′ sites, demonstrating that naturally occurring AG-less sites are independent of downstream splice sites containing the AG dinucleotide. In contrast, all mutations at AG-less splice sites upstream of canonical AG sites to generate canonical AG sites enhanced splicing to upstream mutated splice sites and completely suppressed splicing to downstream canonical sites.

We further investigated whether mutations at branch sites could selectively weaken splice sites. When A to G mutations were introduced simultaneously at the two nearest putative branch sites to the alternative 3′ splice sites in the intron 8 of *RBFOX2*, splicing to the downstream canonical site was preferentially suppressed (Figure 3C). In contrast, A to G mutations at further upstream sites preferentially weakened splicing to the upstream non-canonical splice site. Taken together, our data show that introns containing 3′ dinucleotides other than AG and AC can be spliced by the U2-type spliceosome in a downstream AG-independent manner. Therefore, AG-less splice sites within appropriate sequence contexts must be suitable for the second step of the splicing reaction. The weak upstream non-canonical 3′ splice sites are evolutionarily conserved at least among eutherian mammals for alternative splicing to the downstream in-frame splice sites of *RBFOX* and *CNBP* (Supplementary Figure S10).

### ZRSR2 binds to U12 snRNA and U12-type introns

In contrast to U2AF1, other U2AF1-related splicing factors including ZRSR2 have not been as extensively studied. Although loss-of-function mutations in the *ZRSR2* gene frequently occur in hematologic disorders, the exact roles of ZRSR2 in splicing of U2-type and U12-type introns have not been firmly established. Because an anti-ZRSR2 antibody adequate for CLIP experiments was not available, we generated a knock-in cell line derived from the H1 human ESC line to express a HA-tagged ZRSR2 protein from its natural locus on the X chromosome. We constructed 4 independent CLIP libraries using an anti-HA antibody and obtained 11305226 collapsed reads (Supplementary Figure S11). In addition, we generated 2 independent libraries as a negative control by using an IgG antibody to obtain 4170376 unique collapsed reads.

**Figure 4.**
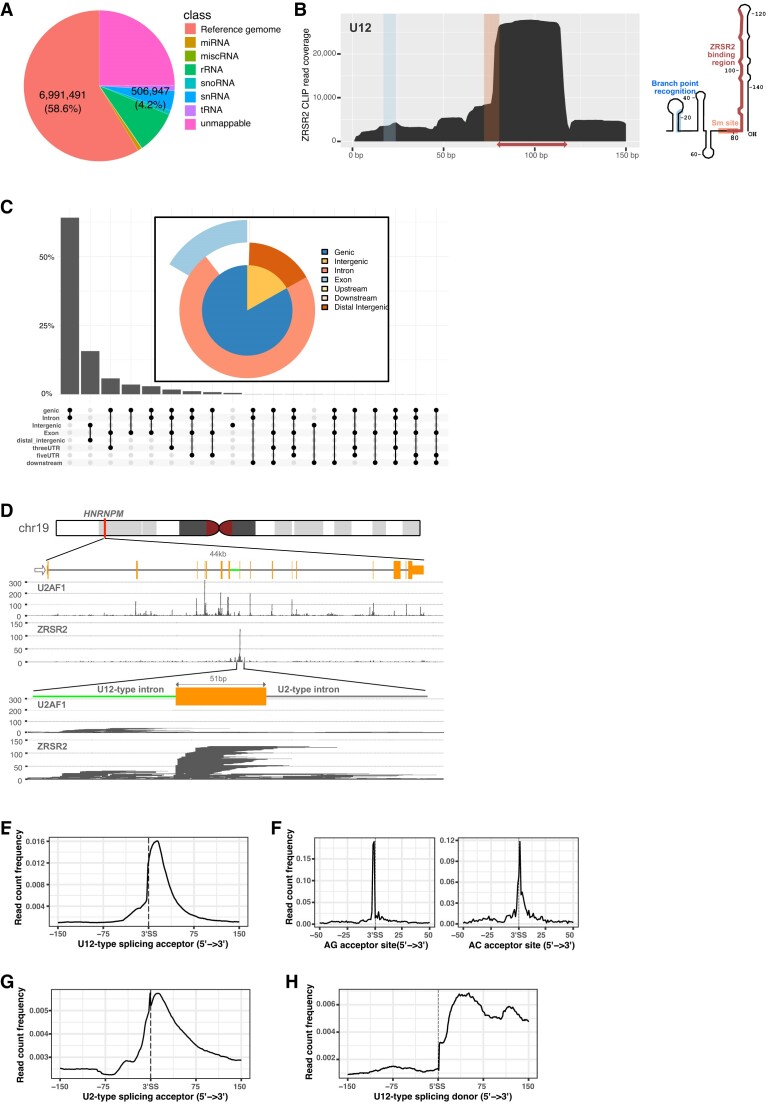
ZRSR2 binds U12 snRNA and U12-type introns. (**A**) Distribution of ZRSR2 CLIP reads on the genome. The pie chart shows the percentages of collapsed reads mapping to abundant non-coding RNAs and the UCSC human reference genome (GRCh37/hg19). See also Supplementary Figure S11 for detailed information. (**B**) Coverage profile on U12 snRNA. The rectangle and the arrow represent the Sm-binding site and the fourth stem-loop structure, respectively. The branch point recognition site, the Sm-binding site and the ZRSR2 binding region are marked on the predicted secondary structure of U2 snRNA. (**C**) ZRSR2 binding site on the human genome. The UpSet plot and the pie chart show the distribution of reads uniquely mapping to the hg19 human genome in the intergenic, intronic, coding regions and UTRs. (**D**) U2AF1 and ZRSR2 binding to the *HNRNPM* mRNA. Reads aligned to the entire locus demonstrate specific binding of ZRSR2 to the U12-type intron and the 3′ exon and U2AF1 to 3′ splice sites of U2-type introns. The U12-type intron is denoted by the green horizontal line and U2-type introns are represented by gray horizontal lines. (**E**) Coverage profile of ZRSR2 CLIP reads around U12-type 3′ splice sites. (**F**) Distribution of 5′ ends of ZRSR2-associated RNAs. Coverage plots using 5′ end nucleotides of reads show the slight difference of distributions at the 3′ ends of two subtypes of U12-type introns. (**G**) Coverage profile of ZRSR2 CLIP reads around U2-type 3′ splice sites. We note that reads covering U2-type splice sites are rare and some U2-type splice sites are close to U12-type introns. (**H**) Coverage profile of ZRSR2 CLIP reads around U12-type 5′ splice sites.

Abundantly expressed noncoding RNAs contributed 16.5% of total CLIP reads and a larger fraction of reads (58.6%) mapped in pre-mRNAs and long non-coding RNAs (Figure 4A). Notably, a significant fraction (3.2%) of ZRSR2 CLIP reads originated from moderately expressed U12, yet a much smaller fraction (1.1%) of reads were derived from all other spliceosomal snRNAs. The contact of ZRSR2 with U12 was constrained to the predicted stem-loop structure immediately downstream of the Sm-binding site, accounting for more than 74% of recovered U12 RNA sequences (Figure 4B).

We next analyzed the locations of ZRSR2 CLIP reads on the transcribed regions of the human genome (Figure 4C). In contrast to results in U2AF1 CLIP, 3′ splice sites were marginally enriched by ZRSR2 CLIP, accounting for just 3.8% of reads mapping in genic regions. Marginal enrichment of 3′ splice sites suggests that the ability of ZRSR2 to make direct contact with general 3′ splice sites is not required for the vital function of ZRSR2, and that binding of ZRSR2 might be restricted to introns having special properties. The ZRSR2 CLIP coverage profile relative to the 3′ splice site shows that enrichment peaking at 15 nt downstream of the annotated 3′ splice site and an abrupt coverage change at the 3′ splice site, which is distinct from the U2AF1 CLIP coverage profile (Supplementary Figure S12). Because ZRSR2 selectively contacts U12 snRNA of the U12-type spliceosome, we analyzed locations of ZRSR2 CLIP reads on the predicted U12-type introns and the U2-type introns. Because of the slightly different lists of putative U12 introns defined by different computational methods, we combined 3′ splice sites of U12-type introns from three different databases, U12DB (version 1.0) (11), MIDB (version 1.0) (32) and IAOD (33), to obtain 840 distinct 3′ splice sites. The CLIP read coverage profile relative to 3′ splice sites of the predicted U12-type introns showed that ZRSR2 CLIP enriched narrow regions peaking at 15 nt downstream of U12-type 3′ splice sites (Figure 4D and E). In contrast, coverage profile of reads from negative control libraries did show enrichment of U12-type splice sites (Supplementary Figure S13). Notably, many ZRSR2 CLIP reads were aligned from the −2 position of the 3′ splice sites of U12-type introns to diverse positions within 3′ exons, which was not observed at the U2-type splice sites.

Most U12-type introns belong to one of two subtypes having either the major GU-AG terminal dinucleotides or the minor AU-AC terminal dinucleotides. While many ZRSR2 CLIP reads mapping on U12-type introns ending with the AG dinucleotide were aligned from the −2 position of 3′ splice sites, many reads mapping on introns ending with the AC dinucleotide were aligned from the first and second positions in 3′ exons (Figure 4F). In contrast, 3′ ends of ZRSR2 CLIP reads mapping on U2-type introns were frequently located at the exact 3′ ends of introns (Figure 4G), suggesting that ZRSR2 might bind excised U2-type introns. Thus, we may conclude that the interactions between ZRSR2 and U2-type introns are extremely rare, and that the rare interactions are distinct from the interactions between U2AF1 and U2-type introns and the interactions between ZRSR2 and U12-type introns.

Since ZRSR2 was copurified with the 18S U11-U12 di-snRNP that recognizes both the 5′ splice site and the BPS of a U12-type intron (34), ZRSR2 might contact the 5′ splice site of U12-type introns. To determine whether ZRSR2 contacts 5′ splice sites, we plotted ZRSR2 CLIP read coverage relative to 5′ splice sites of U12-type introns. The CLIP read coverage profile shows that few reads are aligned with upstream exons of U12-type introns yet there are many CLIP reads aligned with U12-type introns from the first positions of introns (Figure 4H). The enrichment of first nucleotides at 5′ ends of U12-type introns is higher with introns having the AU-AC termini than with introns having the GU-AG termini (Supplementary Figure S14). Because enrichment of the exact 5′ ends of U2-type introns was not observed (Supplementary Figure S15), interactions of ZRSR2 with the 5′ ends of introns appear to be limited to U12-type introns.

### Sites spliced by both the U2-type and U12-type apparatuses are rare

One of the remaining fundamental issues in investigating U12-type introns is to reliably define authentic U12-type introns because many annotated U12-type introns have been supported mainly by computational predictions. Therefore, U12-type introns need to be further supported by experimental evidence. Because 3′ splice sites of predicted U12-type introns were preferentially enriched by ZRSR2 CLIP, we exploited ZRSR2 binding to substantiate 3′ splice sites of U12-type introns. For this purpose, we selected 6113 genomic regions as robust ZRSR2 binding sites, which were covered at least 8 times by reads obtained from ZRSR2 CLIP experiments and wider than 9 nucleotides (Supplementary Table S3). The ZRSR2 binding sites overlapped 413 unique splice site sequences among the 840 putative U12-dependent splice sites, indicating that a large fraction of predicted U12-type splice sites in the databases are *bona fide* U12-type splice sites. If multiple mapping to paralogous genes and pseudogenes was allowed, ZRSR2 binding sites overlapped 440 predicted U12-type sites (Supplementary Table S4). The majority of called peaks overlapping U12-type splice sites were also called by other peak calling tools (Supplementary Figure S16). Moreover, coverage profiles around U12-type 3′ splice sites of peaks called by different calling methods are similar to the coverage profile of ZRSR2 CLIP-seq reads (Supplementary Figure S17). In contrast to U12-type splice sites, ZRSR2 binding sites overlapped just 161 3′ splice sites among the 221 261 annotated U2-type 3′ splice sites (Supplementary Table S5), indicating that binding of ZRSR2 to U2-type splice sites is rare.

To estimate unnoticed U12-type splice sites, we manually examined 5′ splice site and branch point sequences of 225 introns that were enriched by ZRSR2 CLIP but not classified as U12-type introns in the U12-type intron databases yet. We found five putative 3′ splice sites having U12-type consensus sequences located in four different genes (Supplementary Table S6), although the supporting evidence such as Known RefSeq and ESTs is mostly very weak. The rarity of novel U12-type splice sites suggests that most of the highly effective U12-type splice sites might have already been identified by current computational methods. Most interestingly, a 3′ splice site in the *CCDC84/CENATAC* gene could not be classified simply as a U12-type or U2-type site because the site is efficiently spliced to both a U2-type and U12-type sites (Supplementary Figure S18). Enrichment of the 3′ splice site by U2AF1 CLIP, the shift of splicing towards the U2-type 5′ site in cells containing mutations in the gene encoding U4atac and the presence of evolutionarily conserved U2-type and U12-type 5′ splice sites and the U12-type branch site (35,36) strongly support the notion that the site might be an authentic dual-type 3′ splice site in physiological conditions in human cells. Thus, we may expect other splice sites that are used by both the U2- and the U12-dependent splicing systems in physiological conditions if introns have GU-AG terminal dinucleotides.

Since U2AF has been demonstrated not to contact the 3′ splice site in *in vitro* splicing of the U12-type intron of the *NOP2* gene (16), we next surveyed splicing patterns of 101 predicted U12-type 3′ splice sites overlapping U2AF1 binding sites to discover dual-type and misclassified 3′ splice sites. Most of the U2AF1 bound U12-type sites were also enriched by ZRSR2 CLIP except for eight sites that were exclusively enriched by U2AF1 CLIP. Among the U2AF1 bound U12-type sites we identified three 3′ sites in the *DRAM2*, *PFDN5* and *KANSL2* genes to which alternative U2-type and U12-type 5′ sites were effectively spliced in physiological conditions (Supplementary Table S7). In addition to these efficient dual-type sites, we found seven predicted U12-type sites that were occasionally spliced to alternative 5′ sites containing U2-type sequences as well as major U12-type 5′ slice sites, which were supported by an mRNA-seq dataset from H1 cells (GEO: GSE227854) and ESTs. Other 3′ sites enriched by both U2AF1 CLIP and ZRSR2 CLIP were spliced to 5′ sites containing U12-type consensus sequences, indicating that dual-type splice sites are rare among authentic U12-type 3′ splice sites under physiological conditions.

Notably, most U12-type introns enriched by both U2AF1 CLIP and ZRSR2 CLIP are predicted by all computation methods used by three different databases, U12DB, MIDB and IAOD, and U12-type consensus sequences in these introns are mostly conserved across mammalian genomes. In contrast, eight sites exclusively enriched by U2AF1 CLIP were predicted by only one of the three computational methods, indicating these introns marginally satisfy the criteria of a computational method. The sequences bearing similarities to U12-type consensus sequences in the eight introns in the human genome are poorly conserved among mammalian genomes, suggesting that these introns might be U2-type introns. Interestingly, six 5′ sites exhibiting the strongest homology with the U12-type consensus sequence have the AG dinucleotide at the −1 and −2 positions of the 5′ splice sites (Table 1). A previous study showed that there is an evolutionarily conserved bias against G at the −1 position in 5′ exons of predicted U12-type introns having GU-AG termini, but the bias at the −2 position was not studied (33).

**Table 1.** Misclassified U2-type introns. Nucleotides deviate from U12-type consensus sequences are denoted by italic letters

Gene	5′ site	Branch point	Source	Genomic coordinate (hg19)	Strand
*VPS41*	AA|GTA***C***CCTT	T***TT***T***A***AAT	MIDB	chr7:38810855–38812121	−
*MTBP*	GA|GTAT***G***CTT	TC***T***TTAAC	MIDB	chr8:121463563–121466086	−
*LPHN2*	AG|GTATCCT***A***	TC***T***TT***T***AT	U12DB	chr1:82453026–82455340	+
*ELP3*	AG|GTATCCT***G***	T***TT***TTAAT	MIDB	chr8:28017974–28019513	+
*COG6*	AG|GTATCCT***G***	T***T***CTTAAT	MIDB	chr13:40251717–40253674	+
*CSNK1G1*	AG|GTATCCTT	***CT*** CTT***T***AC	MIDB	chr15:64497149–64499707	−
*APPBP2*	AG|GTATCCTT	T***TT***TTA***G***C	MIDB	chr17:58531854–58533656	−
*RTTN*	AG|GTATCCTT	TC***AA***TAAT	MIDB	chr18:67727279–67733064	−
Consensus	**|RTATCCTT	TCCTTRAY			

Vertical lines represent 5′ splice sites.

Among the 47 predicted U12-type GU-AG introns containing G at the −1 position, 26 introns have AG at the −1 and −2 positions, 16 introns have UG, and five introns have GG (Supplementary Table S8). Although introns having AG at the −1 and −2 positions are most common among predicted U12-type G|GU-AG introns, human sequences of the introns showing homology with the U12-type consensus sequences are not conserved among mammalian genomes. Furthermore, none of the predicted U12-type introns enriched by ZRSR2 CLIP have AG at the −1 and −2 positions of 5′ splice sites, supporting the notion that these are not genuine U12-type introns.

In contrast, most 3′ splice sites of predicted U12-type GU-AG introns having the UG or GG dinucleotide at the 5′ splice site were enriched by ZRSR2 CLIP. Moreover, the U12-type consensus sequences on the introns having UG or GG at the −1 and −2 positions are evolutionarily conserved among mammals at 18 of 21 sites, strongly supporting the notion that most of these introns are authentic U12-type introns. Despite the absence of the CG dinucleotide at the −1 and −2 positions of predicted human U12-type introns, we found that four evolutionarily conserved mouse U12-type introns in the *Efl1*, *Morc4*, *Tctn3* and *Oxct1* genes have the CG dinucleotide at the −1 and −2 positions (32). Moreover, human orthologs of introns in the *EFL1* and *TCTN3* genes were enriched by ZRSR CLIP. Thus, we conclude that the AG dinucleotide is particularly incompatible with the U12-type splicing system in evolutionary terms, which is consistent with the presence of the AG dinucleotide at the −1 and −2 positions of most U2-type 5′ splice sites having similarities to the consensus sequence of the U12-type 5′ splice site.

### The upstream AG dinucleotide of U12-type introns is detrimental to splicing

The absence of the AG dinucleotide at the −2 and −1 positions of authentic U12-type introns raises the possibility that AG is especially detrimental to *in vivo* splicing mediated by the U12-type splicing apparatus. To determine the effect of AG on splicing of naturally occurring U12-type introns, we generated three different mini-gene constructs derived from the *EXOSC1*, *RABGGTA* and *SBNO1* genes containing U12-type G|GT-AG introns (Supplementary Figure S2). Then, the U or G nucleotide at the −2 position was replaced by A to generate mutant mini-gene constructs containing AG at the −1 and −2 positions of 5′ splice sites.

**Figure 5.**
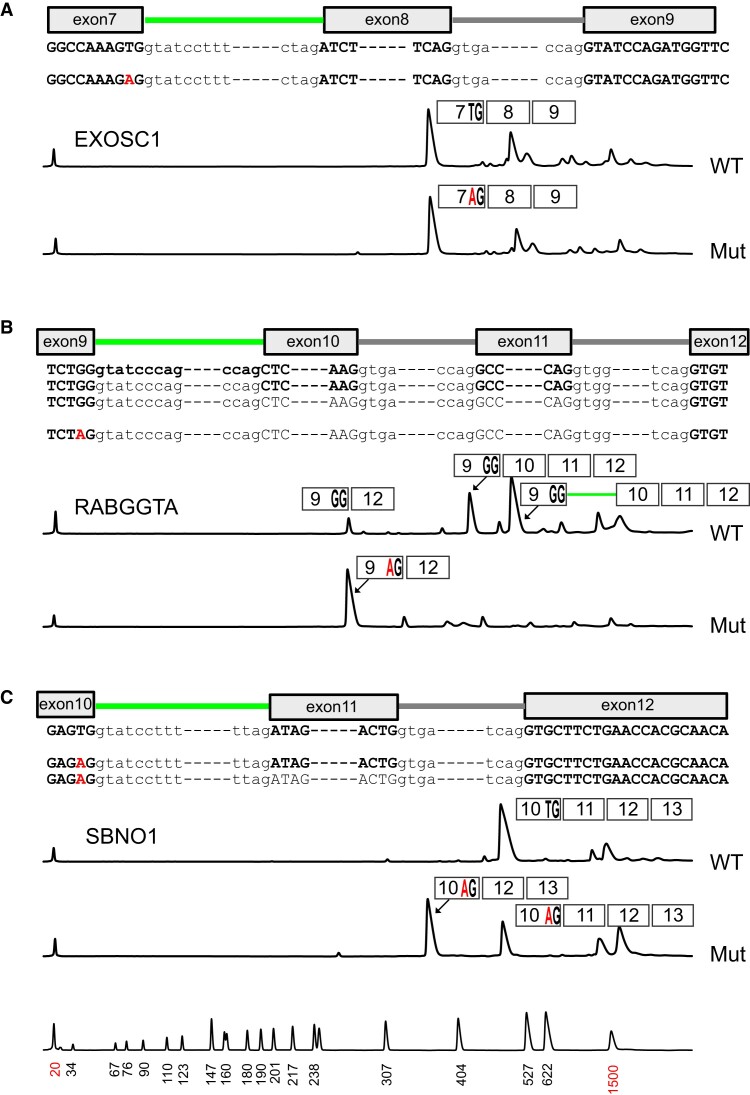
Effect of the AG dinucleotide at the 5′ splice site on selection of the spliceosome class. Spliced RNA products from (**A**) *EXOSC1*, (**B**) *RABGGTA* and (**C**) *SBNO1* mini-gene constructs were amplified by RT-PCR and separated by capillary electrophoresis. The introns between exons 7 and 8 of *EXOSC1*, exons 9 and 10 of *RABGGTA* and exons 10 and 11 of *SBNO1* genes are U12-type introns. The peaks validated by DNA sequencing are represented by rectangles. Nucleotides remaining in spliced RNAs are denoted by bold letters. U2-type and U12-type introns are represented by grey and green lines, respectively. The mutations introduced by nucleotide substitutions at −2 positions of 5′ exons are denoted by red letters. The peaks in the lowest graph represent size makers.

Sequences of spliced RNA products from the transfected wild-type mini-gene constructs showed that U12-type introns were excised at the 5′ and 3′ splice sites identical to the splice sites on pre-mRNAs transcribed from the human genome (Figure 5). Notably, a small fraction of transcripts from the *RABGGTA* mini-gene were spliced between the 5′ splice site of the U12-type intron and a U2-type site by skipping two exons (Figure 5B). In contrast, single nucleotide changes to A at the −2 position altered the splicing patterns of transcripts in various ways. First, the presence of AG affected the splicing of U12-type introns by the U12-type spliceosome in different ways ranging from no effect (*EXOSC1*) to near complete blockage (*RABGGTA*). Second, some mutant U12-type 5′ splice sites were efficiently ligated to U2-type 3′ sites by skipping U12-type 3′ sites. Interestingly, the U12-type intron retained in some spliced RNA products from the wild-type *RABGGTA* mini-gene was almost completely excised in RNA products from the mutant mini-gene construct, demonstrating that the mutant U12-type 5′ splice site containing AG can be more efficiently spliced to a U2-type splice site by the U2-type spliceosome than the wild-type site to the U12-type 3′ site by the U12-type spliceosome. It is striking that just a single nucleotide change from G to A at the −2 position of the U12-type intron in *RABGGTA* can convert the U12-type 5′ splice site to a U2-type site that can be spliced to U2-type 3′ sites in an exclusive manner.

## Discussion

Pre-mRNA splicing depends on the recognition of authentic splice sites among numerous pseudo sites and the selection of correct pairs among a multitude of authentic 5′ and 3′ splice sites. The presence of two distinct splicing systems adds an extra layer of complexity on splice site selection. Although numerous studies on the molecular mechanisms of defining functional splice sites have been conducted, some fundamental questions remain to be addressed. The U2AF1 splicing factor and its family members play a role in defining the 3′ splice sites prior to the first catalytic step of splicing by binding to 3′ splice sites. Here, we present *in vivo* protein-RNA interaction maps of U2AF1 and ZRSR2 to provide insights into their *in vivo* binding specificities and recognition of splice sites by different splicing systems.

### U2AF1 contacts the AG dinucleotide at most binding sites

Although the U2AF heterodimer is one of the most extensively studied splicing factors, the function of each subunit is still debatable. Biochemical studies have suggested that U2AF binds RNA mainly through the U2AF2 subunit that interacts with strong pyrimidine-rich elements (28,37,38), and that introns with weak pyrimidine-rich elements require additional contact between U2AF1 and the AG dinucleotide for efficient U2AF binding and splicing (2,3). However, it is unclear to what extent AG contributes to the *in vivo* recognition of the 3′ splice site by U2AF1 because the AG dinucleotide is also required for the second catalytic step of U2-dependent splicing. In contrast to the highly conserved AG dinucleotide, the less conserved G nucleotide at the + 1 position of the following exon has not been extensively studied as a functional part of the intron-exon boundary.

Guth *et al.* showed that the presence of both subunits of U2AF was required for *in vitro* spliceosome assembly in U2AF-depleted nuclear extracts on a mouse IgM intron containing the most common AG|G 3′ splice site (29). In contrast, the spliceosome was not assembled on a mutant intron containing the AG|C site. Instead of *in vitro* spliceosome assembly, we determined numerous *in vivo* U2AF1 binding sites in intronic regions on pre-mRNAs. Based on the enriched sequences from deep intronic regions, we conclude that U2AF1 contacts the AG dinucleotide in the majority of binding sites, and that [C/U]AGGU and [C/U]AGGA are the most and second most favored sequence motifs, respectively, to which U2AF1 binds. The motifs enriched by U2AF1 CLIP are similar to the most and second most prevalent motifs at annotated 3′ splice sites of U2-type introns, CAG|GU and CAG|GA, respectively.

### Splicing of AG-less U2-type introns is independent of the downstream AG dinucleotide

In contrast to annotated 3′ splice sites, a significant fraction of U2AF1 binding sites in intronic regions lack AG at peaks of U2AF1 binding, demonstrating that the AG dinucleotide is not always required for *in vivo* UV crosslinking of U2AF1 to RNA. Notably, U2AF1 binds AG-less regions associated with long polypyrimidine-rich elements. Because U2AF2 binds pyrimidine-rich elements, U2AF1 associated with U2AF2 might be bought together with the downstream regions of the pyrimidine-rich elements. Nonetheless, almost all known mutations at the AG dinucleotide at the 3′ ends of U2-type GU-AG introns block splicing, supporting the notion that AG is required for splicing in a later step. However, the AG dinucleotide is not always required for splicing of U2-type introns.

AG-less 3′ splice junctions are frequently associated with downstream alternative splice sites. It was previously proposed that the U2-type spliceosome recognizes a downstream canonical 3′ splice site at an early splicing step but chooses an upstream canonical splice site during later steps (39). Similarly, other researchers suggested that U2AF1 binding to downstream AG dinucleotides might exempt initial recognition of upstream 3′ UG splice sites by U2AF1 (30). However, based on mutational analysis, we conclude that the downstream AG dinucleotide is dispensable for splicing to non-canonical sites. Our data show that the excision of introns containing several different kinds of dinucleotides at their 3′ termini is not a unique feature of the U12-type splicing system. The unusual U2-type splice site structures are tightly conserved among mammalian genomes, presumably because of the intrinsically weak non-canonical sites that allow for splicing to downstream canonical 3′ sites.

### ZRSR2 mainly binds U12 snRNA and U12-type introns

Since the structure of ZRSR2 is related to that of U2AF1, ZRSR2 has been presumed to play a role in splicing in a manner similar to that of U2AF1. However, the exact functions of ZRSR2 in splicing of U2-type and U12-type introns have been controversial. Based on the observation that ZRSR2 was associated with the U2AF heterodimer in nuclear extracts, ZRSR2 was proposed to be a component of a larger U2AF complex (8). *In vitro* splicing assays suggest that ZRSR2 is required both for initial recognition of the 3′ splice site on a U12-type intron and for the second catalytic step of splicing of a U2-type intron (16). However, the cryo-EM structure of the human U2-type spliceosome, in the catalytically activated form for the second catalytic step of splicing, does not contain ZRSR2 (40,41). The loss of ZRSR2 activity in cell lines leads to defects confined to U12-type intron splicing, suggesting that ZRSR2 plays either a limited or redundant role in *in vivo* splicing of U2-type introns (14). Furthermore, ZRSR2 has been found in the human 18S U11/U12 di-snRNP but not in the U2-dependent spliceosome (34), consistent with a limited, if any, role of ZRSR2 in splicing of U2-type introns.

In this study, we demonstrate that ZRSR2 exclusively binds 3′ splice sites of U12-type introns in pre-mRNAs expressed in human ESCs, which is analogous to U2AF1 binding to U2-type 3′ splice sites. For all similarity in the domain structures of U2AF1 and ZRSR2, ZRSR2 binding sites suggest that there are mechanistic differences in the way U2AF1 and ZRSR2 facilitate splicing of U2-type and U12-type introns, respectively. First, ZRSR2 binding peaks in 3′ exons of U12-type introns and 5′ ends of enriched RNAs by ZRSR2 CLIP are located at around U12-type 3′ splice sites. In contrast, U2AF1 binding peaks around U2-type 3′ splice sites. Second, while U2AF1 does not contact snRNAs, ZRSR2 specifically contacts U12 snRNA. Furthermore, the enrichment of exact 5′ ends of U12-type introns suggests that ZRSR2 remains on U12-type introns after the first catalytic step, although ZRSR2 was not contained in the cryo-EM structure of the minor B^act^ complex (42). ZRSR2 seems to leave the U12-type spliceosome before the completion of the second catalytic step because 3′ ends of CLIP enriched RNAs do not ends at 3′ ends of U12-type introns. In contrast, U2AF1 does not bind to 5′ ends of U2-type introns. While *in vitro* UV-crosslinking and *in vitro* splicing experiments indicated that ZRSR2 specifically contacts 3′ splice sites of a U2-type intron as well as a U12-type intron (16), *in vivo* splicing of U2-type introns was largely unaffected by loss-of-function mutations in ZRSR2 (14). In sum, confined binding of ZRSR2 to U12 snRNA and U12-type introns is in line with the notion that ZRSR2 is a dedicated factor for splicing of U12-type introns *in vivo*.

### RNA elements important for the choice of the spliceosome class

Splice sites have been exclusively classified into U2-type and U12-type sites since the discovery of U12-type introns and the U12-type spliceosome. Such dichotomous classification of splice sites is adequate in most cases because naturally occurring U12-dependent splice sites are usually incompatible with the U2-type spliceosome. However, it has long been known that some introns can be removed by both the U2-type and the U12-type spliceosomes (43).

Some U2-type introns have sequences similar to U12-type 5′ splice site consensus sequences, suggesting that the highly conserved U12-type 5′ splice site consensus sequence *per se* might be a legitimate U2-type splice site. Moreover, the U2 snRNP can recognize the U12-type BPS because the U2-type BPS has the highly degenerate sequence motif including the highly constrained U12-type BPS consensus sequence. Nonetheless, only a small number of 3′ splice sites of naturally occurring U12-type GU-AG introns are spliced by both the U2-type and U12-type spliceosomes. This suggests there may be additional elements that can either stimulate splicing by the U12-type spliceosome or suppress splicing by the U2-type spliceosome.

In this study, we surveyed favored and disfavored nucleotides in exon termini that flank U12-type introns. Most strikingly, no AG dinucleotides were found at the very ends of 5′ flanking exons of any validated naturally occurring U12-type introns with GU-AG terminal dinucleotides. It appears that all predicted human U12-type introns having the AG|GU 5′ splice site are misclassified U2-type introns (Table 1). In line with the absence of AG, Moyer *et al.* discovered the evolutionarily conserved bias against −1 G only in U12-type introns having GU-AG termini (33). In the present study, we demonstrate that, at the extreme, the introduction of the incompatible AG dinucleotide with naturally occurring U12-type introns converts a U12-type 5′ splice site to a U2-type site that is exclusively removed by the U2-type spliceosome.

The strong preference for AG flanking U2-type 5′ splice sites has been attributed to pairing of AG with CU at positions 8 and 9 on the U1 snRNA (44,45). The absence of flanking AG at the GU-AG U12-type 5′ splice sites cannot be ascribed to similar pairing between the 5′ splice sites and the U11 snRNA because U11 is expected to interact with nucleotides at +5 to +9 positions of 5′ splice sites. The presence of AG at the −1 and −2 positions of the U12-type 5′ consensus sequence may allow for recognition of the sites by the U1 snRNP with better efficiency because the AG dinucleotide appears to play a crucial role for efficient recognition of U2-type 5′ splice sites showing weak homology with the U2-type consensus sequence (46). The occupation of the 5′ splice site by the U1 snRNP may inhibit correct pairing between U12-type 5′ and 3′ splice sites. The bias against G at the −1 position of 5′ splice sites of U12-type introns is likely to be a consequence of natural selection to prevent the U1 snRNP from binding to U12-type splice sites. We expect that in most cases recognition of splice sites by both the U2-type and U12-type spliceosomes might be detrimental to correct splicing and, therefore, expression of host genes. In contrast to GU-AG introns, U12-type AU-AC introns seem to be poorly recognized by the U2-type spliceosome in any sequence context, so the evolutionary pressure against the AG dinucleotide at the −1 and −2 positions may be much weaker.

On the 3′ side of U12-type introns, intron-exon boundaries have the CA[G/C]|AU motif (Supplementary Figure S19) instead of [C/U]AG|GU that U2AF1 preferentially binds (Supplementary Figure S7 and S8). Unlike the U2-type intron, the C or U nucleotide is more frequently found at the + 1 position of the downstream exon of the U12-type intron than the G nucleotide. The bias for A at the + 1 position of the 3′ exon is in line with the proposition that U2AF1 is not involved in recognition of U12-type 3′ splice sites. Different models could explain the preference for the distinct terminal AU dinucleotide on the 3′ exons of U12-type introns. First, A[G/C]|AU might be preferentially recognized by ZRSR2, analogous to preferential binding of U2AF1 to the AG|GU motif. Second, pre-mRNAs containing the motifs are better substrates for the second catalytic step of splicing. Third, the U2-type 3′ site motif is detrimental to splicing by the U12-type spliceosome.

To our knowledge, U12-type introns in databases have been predicted using the weight matrices for intronic consensus sequences. In this study, we found that computational prediction of U12-type introns is so effective that few, if any, novel U12-type introns are yet to be identified in the human genome. However, information content in the terminal dinucleotide pair of the intron and U12-type consensus sequence motifs located in intronic regions might not be sufficient to exclusively define a splice site pair by the U12-type spliceosome. In this study, we showed that nucleotides in exon could be parts of RNA elements playing a role in the splicing of U12-type introns exclusively by the U12-type spliceosome. The computational prediction of U12 introns with revised weight matrices for the consensus sequence motifs expanded to exonic regions may improve the identification of authentic U12-type introns and detrimental mutations around splice sites of U12-type introns.

## Supplementary Material

gkad1180_supplemental_files

## Data Availability

The raw and processed data are available in the Gene Expression Omnibus under accession code GSE203531.
