# Supplementary material for: Global analysis of binding sites of U2AF1 and ZRSR2 reveals RNA elements required for mutually exclusive splicing by the U2- and U12-type spliceosome
Source: Nucleic Acids Res. 2023 Dec 13;52(3):1420–34. doi: 10.1093/nar/gkad1180 (PMC10853781; doi:10.1093/nar/gkad1180)
Supplement: gkad1180_supplemental_files [file gkad1180_supplemental_files.zip › Legends Supplementary Tables.docx]

Supplementary Table S1. Oligonucleotides used in this study.

Supplementary Table S2. Non-canonical alternative splice sites.

Supplementary Table S3. Unique ZRSR2 binding sites identified by ZRSR2 CLIP-seq.

Supplementary Table S4. U12-type 3’ splice sites overlapping ZRSR2 binding sites.

Supplementary Table S5. U2-type 3’ splice sites overlapping ZRSR2 binding sites.

Supplementary Table S6. Novel U12-type 3’ splice sites overlapping ZRSR2 binding sites.

Supplementary Table S7. Putative dual-type 3’ splice sites.

Supplementary Table S8. U12-type 5’ splice sites having G at the -1 position.
