# Supplementary material for: Global analysis of binding sites of U2AF1 and ZRSR2 reveals RNA elements required for mutually exclusive splicing by the U2- and U12-type spliceosome
Source: Nucleic Acids Res. 2023 Dec 13;52(3):1420–34. doi: 10.1093/nar/gkad1180 (PMC10853781; doi:10.1093/nar/gkad1180)
Supplement: gkad1180_supplemental_files [file gkad1180_supplemental_files.zip › Supplementary_Data_Figures_revised_2.pdf]

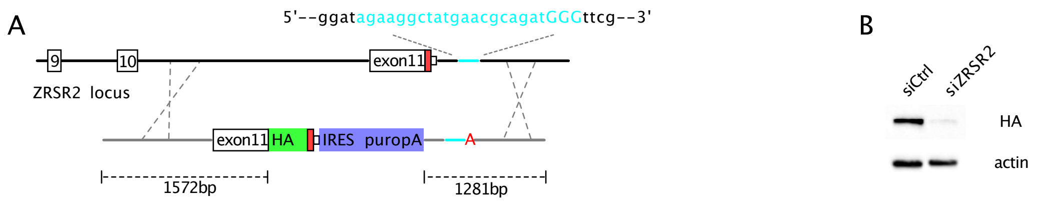

Supplementary Figure S1. Generation of the endogenous HA3-tagged *ZRSR2* allele. (A) Schematic of targeting strategy. The target sequence of sgRNA is denoted in cyan and the protospacer adjacent motif (PAM) is capitalized. The PAM sequence (GGG) is replaced with A (denoted in red) in the donor construct. A HA3-tag was inserted before the stop codon (red rectangle). (B) The validation of *ZRSR2* targeting. The identity of HA-tagged ZRSR2 was verified by treating cells with siZRSR2 (Dharmacon, SMARTPool ON-TARGETplus siRNA).

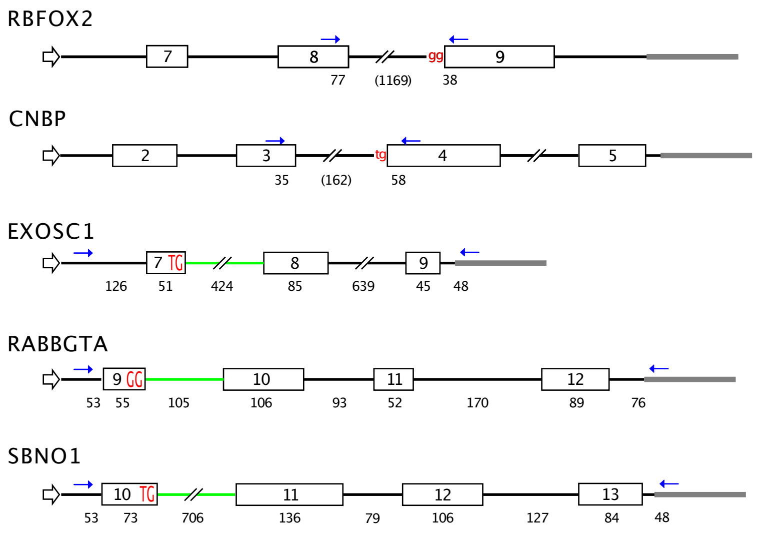

Supplementary Figure S2. Schematics of the mini-gene constructs and primer landing site sequences. The rectangles and bold lines represent exons and introns, respectively. U12-type introns are denoted by green lines. The arrows represent PCR primer landing sites.

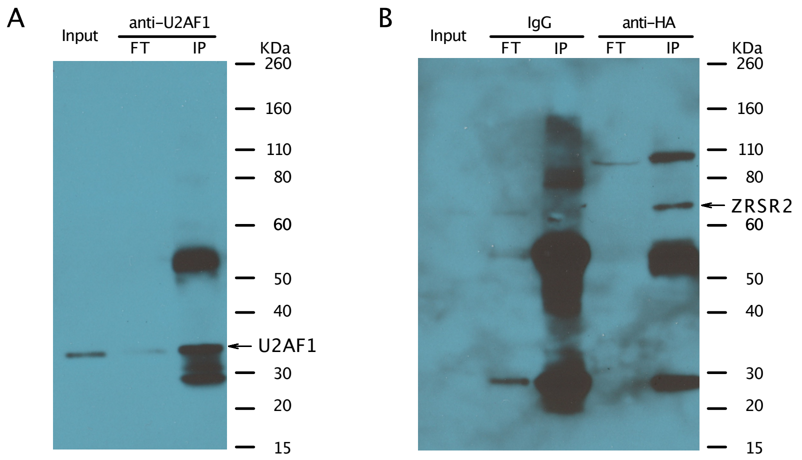

Supplementary Figure S3. Immunoprecipitation of U2AF1 and HA3-tagged ZRSR2. Western blot was carried out to validate immunoprecipitation under CLIP conditions. Specifically enriched protein bands are marked by arrows. Regions from 10 kDa above protein bands were excised to recover crosslinked RNA fragments. Expression levels of endogenous ZRSR2 are much lower that those of U2AF1. The upper band above ZRSR2 might be antibodies that were not sufficiently reduced.

A

Replicate1	Replicate2	Replicate3	Replicate4	IgG control
3,279,014	9,682,232	26,001,963	26,990,225	4,737,076

65,953,372

rRNA: 867,094
snRNA: 362,016
snoRNA: 120,548
tRNA: 234,135
miscRNA: 86,130
miRNA: 714,235
Reference genome: 49,157,741
RefSeq: 394,470
unmappable: 14,017,003

rRNA: 261,038
snRNA: 222,633
snoRNA: 40,776
tRNA: 33,384
miscRNA: 9,501
miRNA: 31,539
Reference genome: 2,833,228
RefSeq: 137,388
unmappable: 1,167,589

B

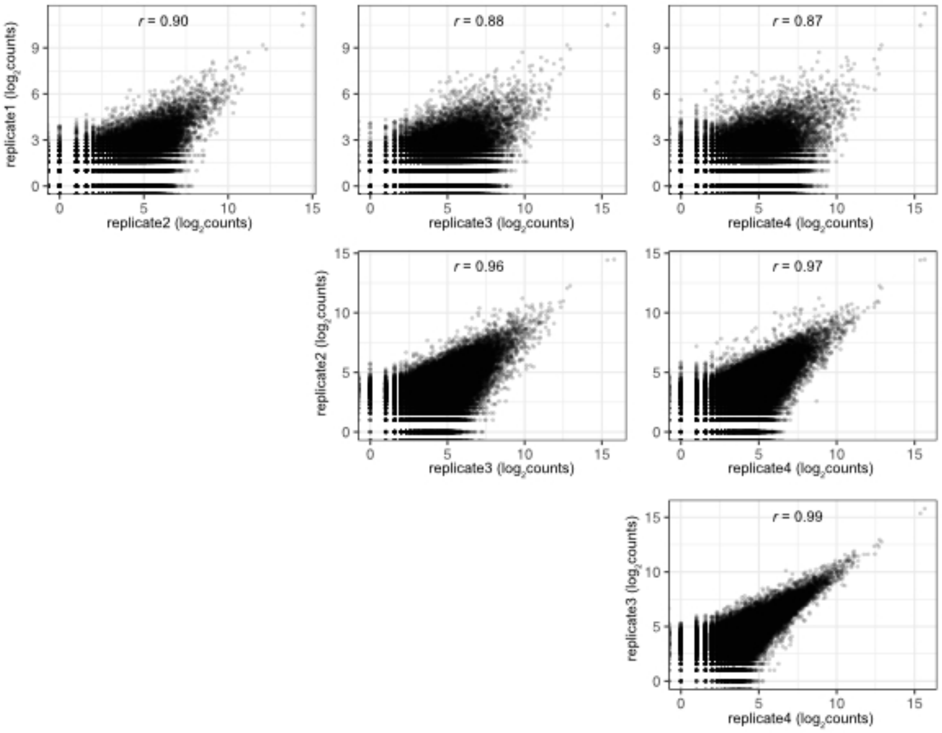

Supplementary Figure S4. (A) Distribution of U2AF1 CLIP-seq reads. (B) Reproducible enrichment of U2AF1 CLIP-seq reads. Each scatter plot shows read counts of indicated experimental replicates overlapping peaks (162,668 sites) and the Pearson correlation coefficient.

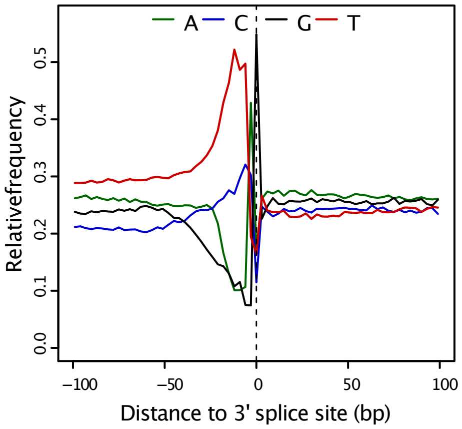

Supplementary Figure S5. Frequency of single nucleotides relative to the annotated 3’ splice sites.

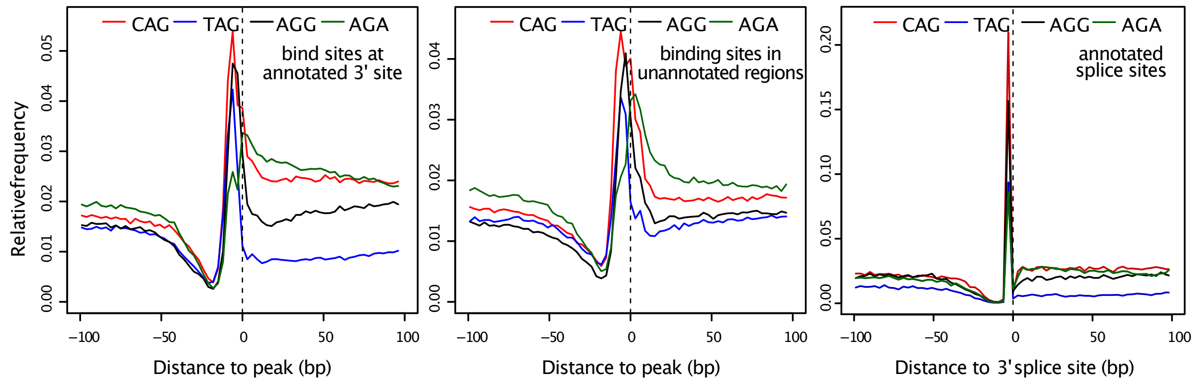

Supplementary Figure S6. Frequency of the most common trimer motifs relative to U2AF1 binding peaks and the annotated 3’ splice sites.

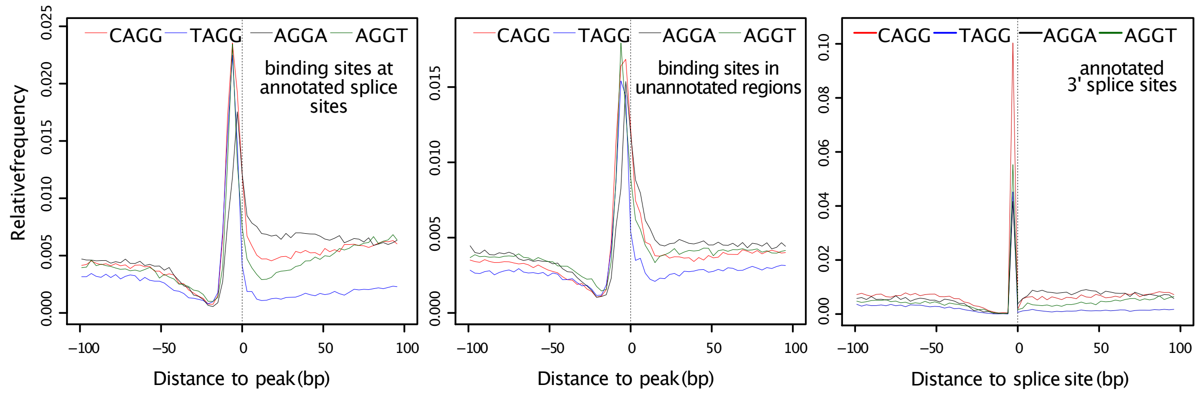

Supplementary Figure S7. Frequency of the most common tetramer motifs relative to U2AF1 binding peaks and the annotated 3’ splice sites.

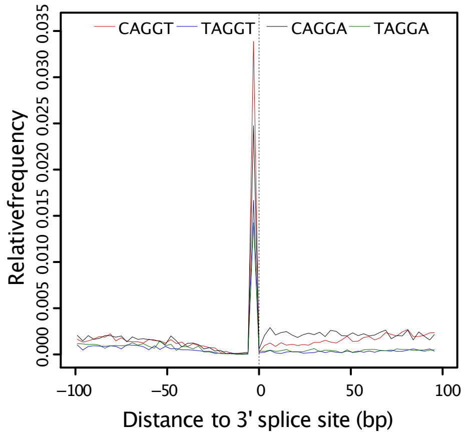

Supplementary Figure S8. Frequency of the most common pentamer motifs relative to the annotated 3’ splice sites.

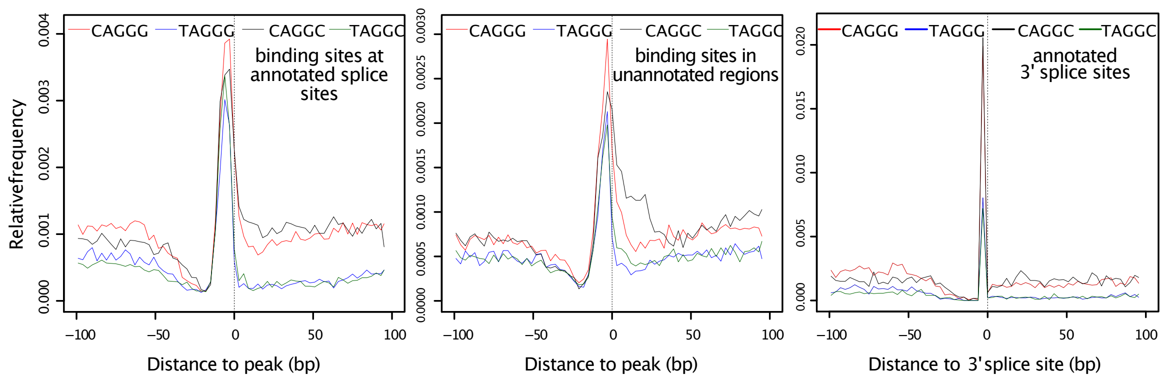

Supplementary Figure S9. Frequency of pentamer motifs relative to U2AF1 binding peaks and the annotated 3’ splice sites.

RBFOX2

```
                                                 ▼            ▼
Human       TGAATGCAAGTTGAAATTTCTGTCATATTTTGGGTCTCCCTTTAGTTCCTGGCTTCCCTT
Rhesus      TGAATGCAAGTTGAAATTTCTGTCATATTTTGGGTCTCCCTTTAGTTCCTGGCTTCCCTT
Mouse       TGAATGCCAGTTGAAATTTCTGTCATATTTTGGGTCTCCCTTTAGTTCCTGGCTTCCCTT
Elephant    TGAATGCAAGTTGAAATTTCTGTCATATTTTGGGTCTCCCTTTAGTTCCTGGCTTCCCTT
Dog         TGAATTCAAGTTGACATTCCTGTCATATTTTGGGTCTCCCTTTAGTTCCTGGCTTCCCTT
            ***** * ****** *** *****************************************
```

CNBT

```
                                        ▼  ▼  ▼
Human       TCTTGGCCTGTTTCTTTTCCTTATTGTTGAAGCCTGCTATAACTGCGGTAGAGGTGGCCA
Rhesus      TCTTGGTCTGTTTCTTTTCCTTATTGTTGAAGCCTGCTATAACTGCGGTAGAGGTGGCCA
Dog         TCTTGGTCTGCTTCTTTTCCTTATTGTTGAAGCCTGCTATAACTGCGGTAGAGGTGGCCA
Mouse       TCTTGGTCTGCTTCTTTTCCTTATTGTTGAAGCCTGCTATAACTGCGGTAGAGGTGGCCA
Elephant    TCTTGGTCTGCTTCTTTTCCTTATTGTTGAAGCCTGCTATAACTGCGGTAGAGGTGGTCA
Platypus    TCTTGGTCTGGTTCTTCTCCTTATTGTTGAAGCCTGCTATAACTGCGGTAGAGGTGGCCA
Chicken     TCTTGTTTGGTATGTTTTTCTTATTGTGGAAGCCTGCTATAACTGCGGTAGAGGTGGCCA
            *****    *  * ** * ******** ***************************** **
```

Supplementary Figure S10. Conservation of upstream sequences of non-canical 3' splice sites. The inverted triangles represent splice sites and nucleotides not conserved are marked by bold letters.

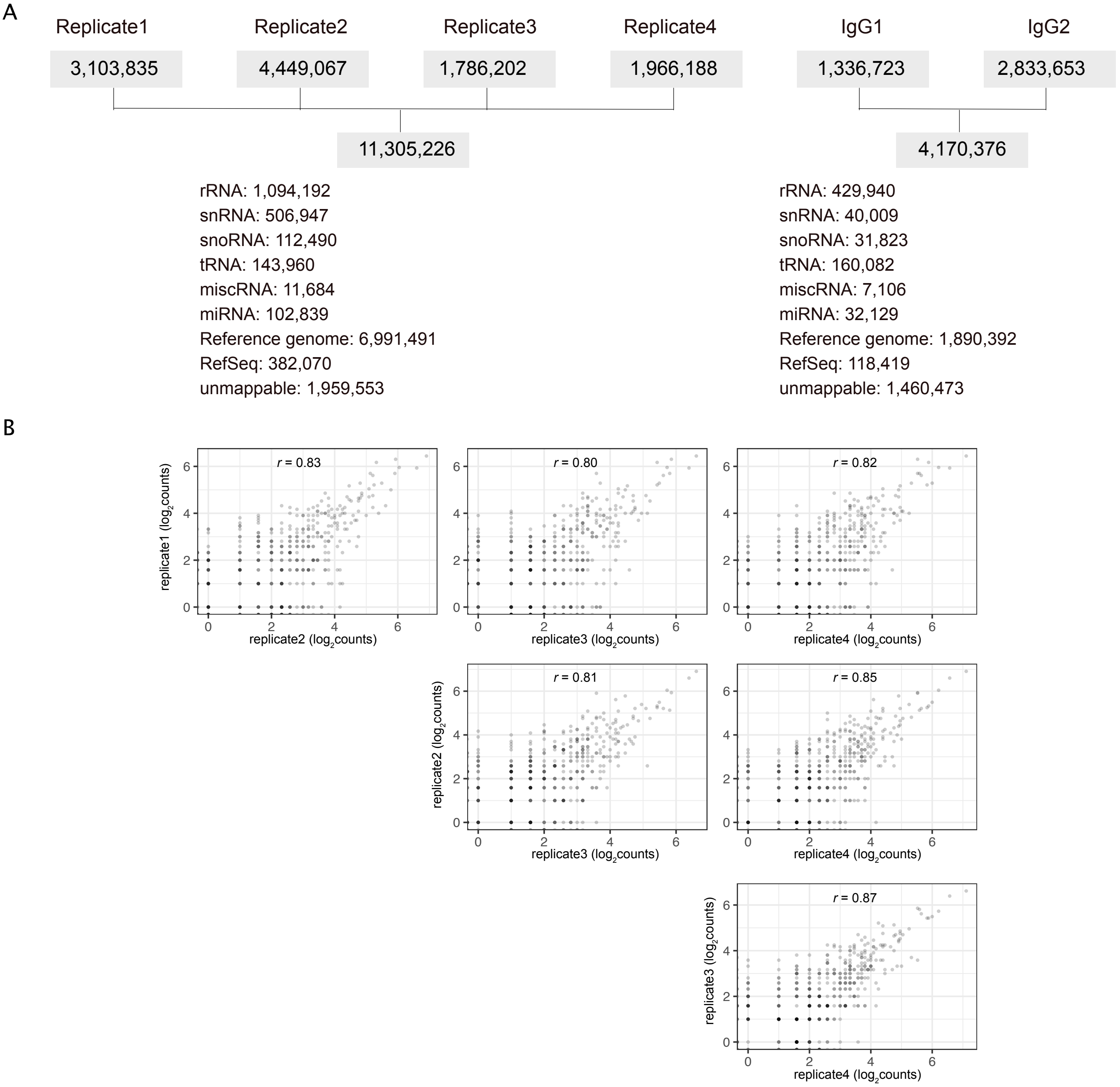

Supplementary Figure S11. (A) Distribution of ZRSR2 CLIP-seq reads. (B) Reproducible enrichment of ZRSR2 CLIP-seq reads. Each scatter plot shows reads counts of indicated experimental replicates overlapping peaks that covering annotated U12-type 3' splice sites.

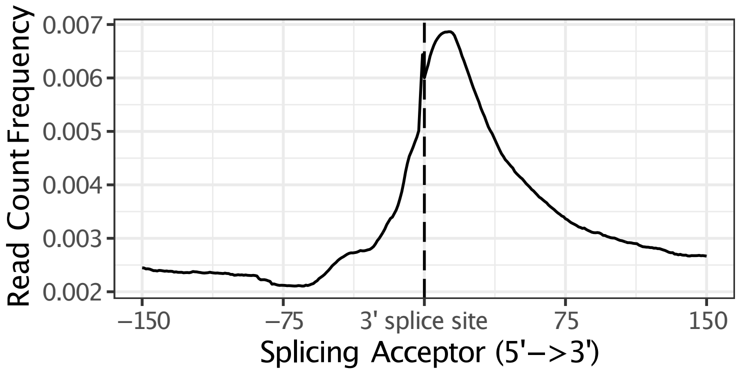

Supplementary Figure S12. Coverage profile of ZRSR2 CLIP reads around annotated 3’ splicing sites.

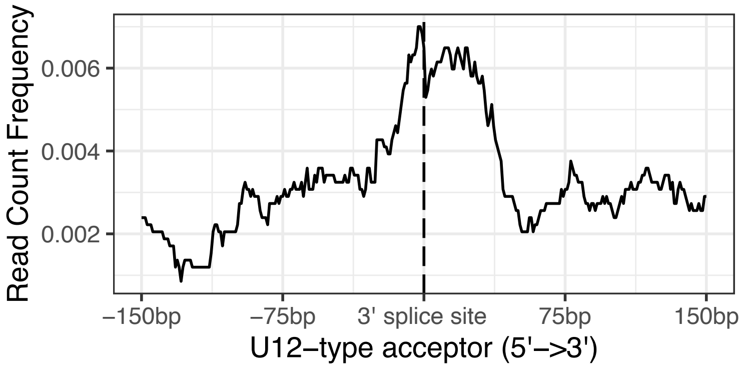

Supplementary Figure S13. Coverage profile of IgG CLIP reads around annotated U12-type 3’ splicing sites.

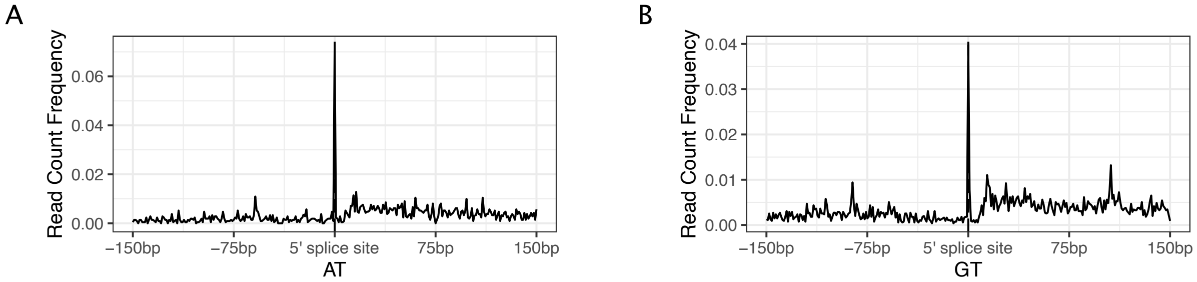

Supplementary Figure S14. Distribution of 5' ends of ZRSR2-associated RNAs over U12-type slice donor sites. Coverage profiles of ZRSR2 CLIP reads around annotated U12-type 5’ splicing sites starting with (A) the AT dinucleotiede and (B) the GT dinucleotide show that the enrichment of first nucleotides at 5’ ends of U12-type introns having the AU-AC termini is higher than introns having the GU-AG termini.

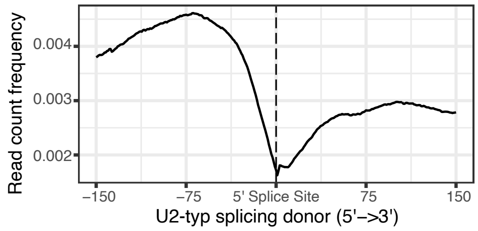

Supplementary Figure S15. Coverage profile of ZRSR2 CLIP reads around U2-type 5’ splicing sites.

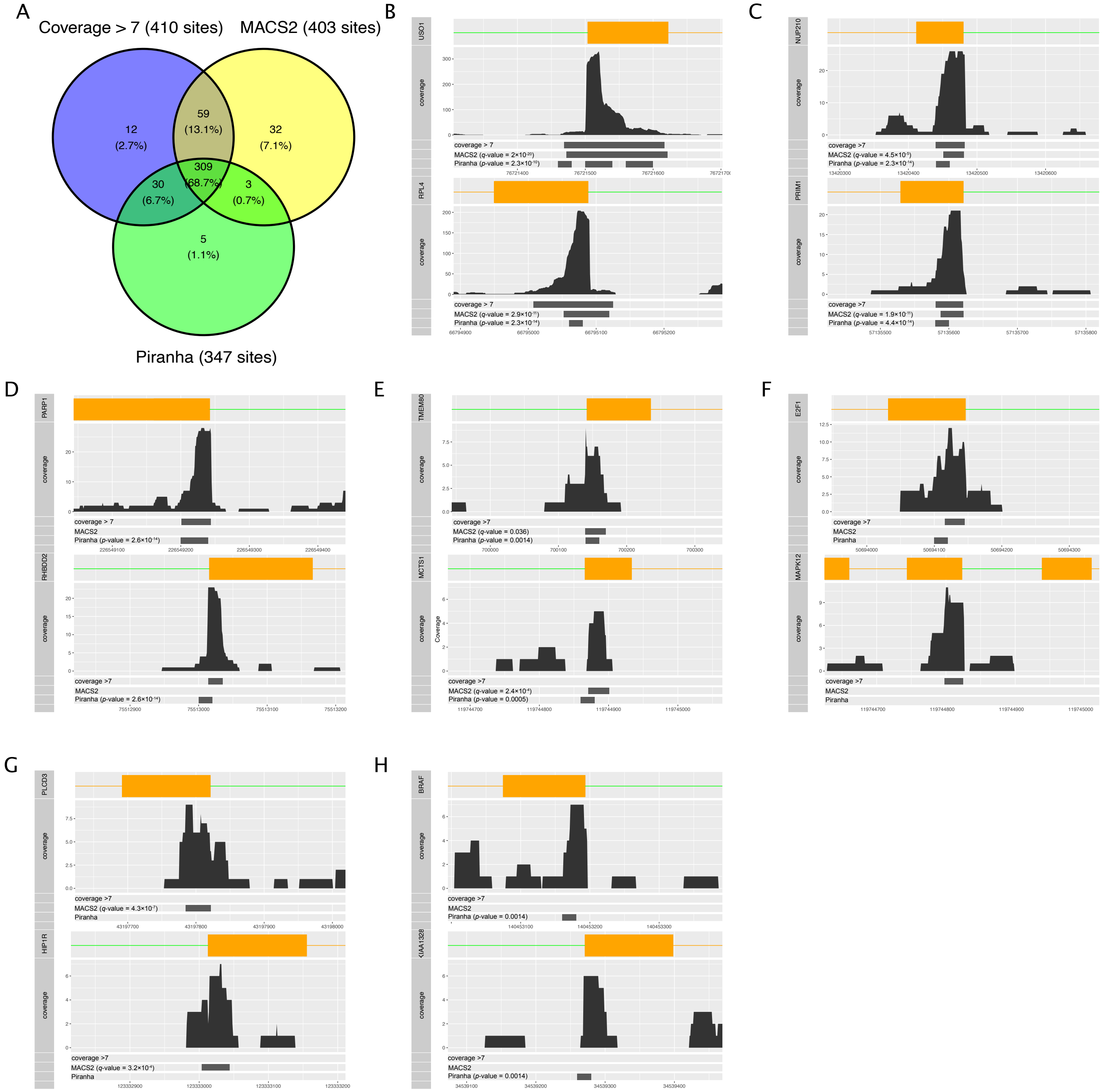

Supplementary Figure S16. U12-type 3' splice sites called by different peak call methods. (A) Venn diagram of U12-type 3' splice sites (-10 to +20) overlapped by peaks called by coverage cutoff, MACS2 and Piranha. Splice sites that overlap were reduced to a single site. ZRSR2 binding sites were defined by regions covered at least 8 times by ZRSR2 CLIP reads and wider than 3 nt (7,492 sites (182,683 nt)). The FDR for the cutoff value 8 for ZRSR2 CLIP is slightly higher than 1.0 x 10^{-4}, which was calculated by using the peakCutoff function of the chipseq package in the Bioconductor repository. The peakCutoff function assumes a Poisson noise distribution that is estimated from the frequency of singleton and doubleton islands. MACS2 and Piranha installed in the Galaxy community hub were used with parameters -t --name ZRSR2_ sorted.bam --format BAM --gsize 2700000000 --keep-dup 1 --d-min 20 --buffer-size 100000 --bdg --qvalue 0.05 --nomodel --extsize 30 --shift 0 for MACS and-s -p 0.1-b 20 -u 1 -r -d ZeroTruncatedNegativeBinomial -n ./ZRSR2_sorted_bam -o ./piranha.out for Piranha, respectively. With these parameters, MACS2 called 3,605 peaks (160,053 nt) and Piranha called 11,641 peaks (264,501 nt). The numbers of peaks and sums of regions defined by peak calling methods are comparable. (B) Representative 3' splice sites defined by all peak calling methods. ZRSR2 CLIP-seqs reads aligned without any mismatched were used for peak calling and covergage profiles. Regions defined by peak calling methods are denoted by gray rectangels. (C) 3' splice sites called by coverage cutoff and MACS2. Many peaks called by Piranha do not overlap exact 3' splice sites. (D) Peaks called by coverage cutoff and Piranha. (E) Peaks called by MACS2 and Piranha. (F) Peaks called by the coverage cutoff method only. (G) Peaks called by MACS2. (H) Peaks called by Piranha. U12-type introns are marked by green lines.

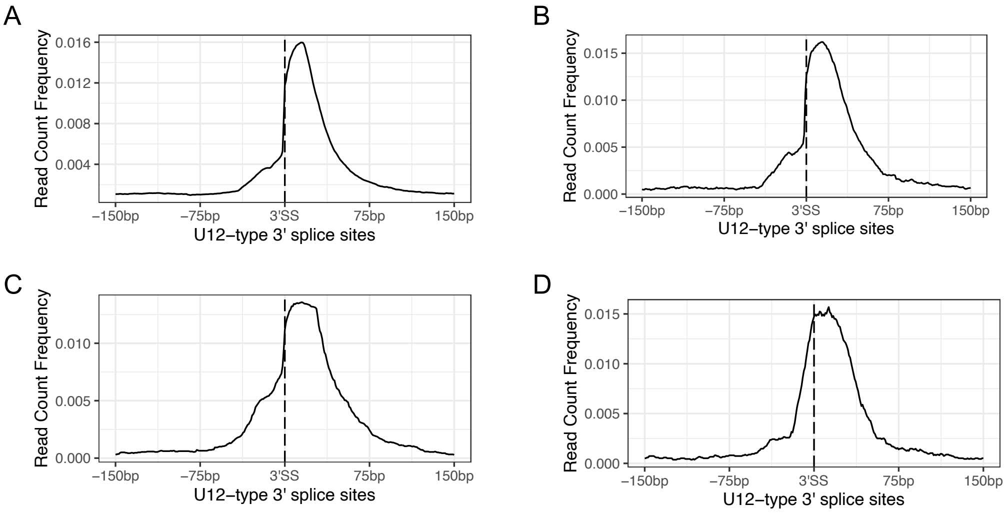

Supplementary Figure S17. Coverage profiles of CLIP reads and peaks called. Coverage profiles of (A) ZRSR2 CLIP-seq reads, (B) peaks defined by coverage cutoff higher than 7, (C) peaks called by MACS2 and (D) peaks called by using Piranha. Because MACS2 does not use strand information, strand information was added after peak calling.

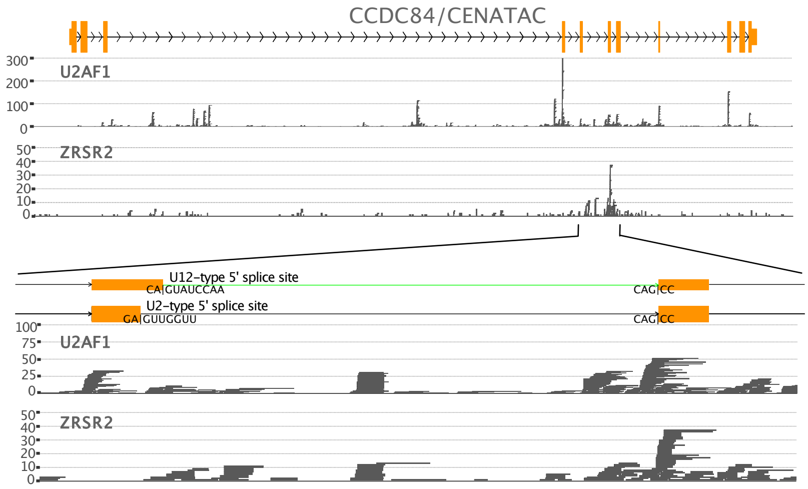

Supplementary Figure S18. U2AF1 and ZRSR2 binding to the *CCDC84/CENATAC* mRNA. The U12-type intron is represented by a green horizontal line and U2-type introns are denoted by black horizontal lines.

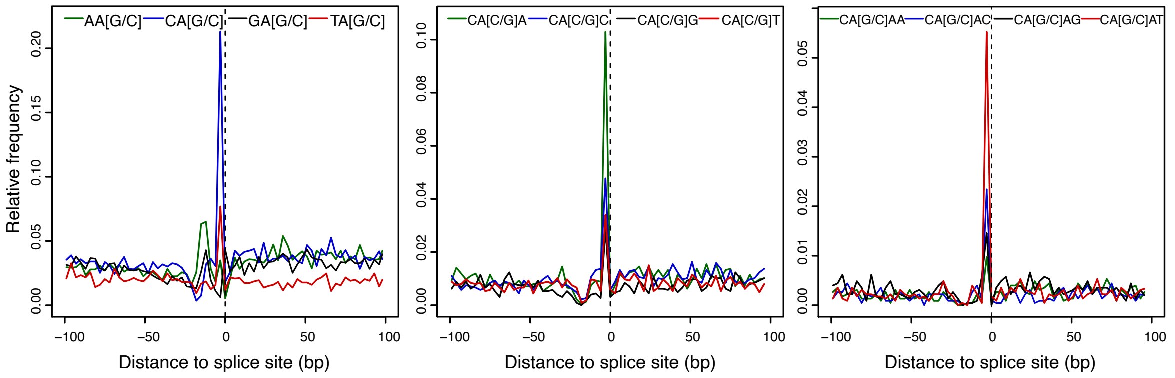

Supplementary Figure S19. Frequency of the most common trimer, tetramer and pentamer motifs relative to the annotated U12-type 3' splice sites.
